# A sequential Monte Carlo algorithm for inference of subclonal structure in cancer

**DOI:** 10.1371/journal.pone.0211213

**Published:** 2019-01-25

**Authors:** Oyetunji E. Ogundijo, Kaiyi Zhu, Xiaodong Wang, Dimitris Anastassiou

**Affiliations:** 1 Department of Electrical Engineering, Columbia University, New York, NY, United States of America; 2 Department of Systems Biology, Columbia University, New York, NY, United States of America; Janssen Research and Development, UNITED STATES

## Abstract

Tumors are heterogeneous in the sense that they consist of multiple subpopulations of cells, referred to as subclones, each of which is characterized by a distinct profile of genomic variations such as somatic mutations. Inferring the underlying clonal landscape has become an important topic in that it can help in understanding cancer development and progression, and thereby help in improving treatment. We describe a novel state-space model, based on the feature allocation framework and an efficient sequential Monte Carlo (SMC) algorithm, using the somatic mutation data obtained from tumor samples to estimate the number of subclones, as well as their characterization. Our approach, by design, is capable of handling any number of mutations. Via extensive simulations, our method exhibits high accuracy, in most cases, and compares favorably with existing methods. Moreover, we demonstrated the validity of our method through analyzing real tumor samples from patients from multiple cancer types (breast, prostate, and lung). Our results reveal driver mutation events specific to cancer types, and indicate clonal expansion by manual phylogenetic analysis. MATLAB code and datasets are available to download at: https://github.com/moyanre/tumor_clones.

## Introduction

In most cases, tumors develop from a single population of cells. Accumulated somatic mutations confer selective advantages to the cells in this population over others [1], and then this population of cells continues to proliferate. As more somatic mutations are acquired, some tumor cells gain further survival advantages, which leads to an expansion from a single population to multiple subpopulations. As a result, tumors are heterogeneous in nature [2, 3] and contain multiple subpopulations of cancerous cells, each with a unique mutational profile [4–6], referred to as tumor subclones [2, 7, 8]. The importance of analyzing the tumor subclonal structure and evolutionary progress has been recognized, considering the potential of elucidating the underlying mechanisms of cancer progression, metastatic spread and therapy response [9–11].

Characterizing tumor heterogeneity with subclonal structure, using next-generation sequencing (NGS) data is a well-studied problem [12], and various computational methods have been proposed for estimating the subclonal structure in the tumor samples [13–17]. Some methods approach this estimation problem by first grouping the mutations into clusters, and then performing phylogenetic analysis to obtain the mutational profiles of the various distinct subclones in the samples [14–17]. A more direct approach bypasses the clustering stage by modeling, in straightforward manner, the NGS data with a feature allocation model [13, 18–20]. Basically, with this setup, the problem is reduced into a form of matrix factorization [21], where the observed variant allele frequency (VAF) is deconvolved into matrices of genotypes of subclones and the proportion of genotypes in the samples [13, 18, 20]. However, methods in this category are faced with several issues, such as the assumption that the number of subclones have to be fixed before analysis [13, 19], and the fact that only a few mutations can be analyzed [19].

Here, we propose an algorithm for estimating the number, genotypes and the proportion of subclones, employing a more general model that better explains the inherent heterogeneity in tumor samples by allowing more categories for the genotypes, so as to capture the three possible genotypes in a diploid individual. Specifically, 0 for homozygous wild-type, 0.5 for heterozygous mutant and 1 for homozygous mutant. Our approach, which is based on the state-space formulation of the feature allocation model, employs the SMC [22–24] algorithm for estimating the model parameters. The proposed SMC algorithm takes advantage of the categorical Indian buffet process (cIBP) [20], a sequential procedure that describes the prior distribution of the general (*Q* + 1)-ary categorical matrix, in modeling the genotypes of subclones. Because the proposed algorithm processes the observed VAF data sequentially, it offers the flexibility of being able to handle any number of mutations without encountering computational issues. More specifically, SMC, a powerful recursive filtering algorithm [21, 25, 26], computes, in a flexible manner, the posterior probability density function (PDF) of the hidden state every time a measurement is observed, approximating the posterior distributions of the variables of interest with a set of properly weighted samples, which we will refer to as particles to distinguish between random samples from a distribution and tumor samples.

Over the simulated datasets, we compare our algorithm with BayClone [20], a Markov chain Monte Carlo (MCMC) based algorithm, often employed when estimating model parameters in tumor heterogeneity [19], and Clomial [13], an expectation maximization (EM) based algorithm. Similar to the our modeling method, BayClone considers the three possible genotypes in a diploid individual. Although the modeling approach in Clomial only considers homozygous wild-type and heterozygous mutant (a common modeling consideration in the analysis of tumor heterogeneity [19]), it employs EM, a different inference algorithm, to estimate the model parameters. Invariably, our simulations compare the performance of three different algorithms: SMC, MCMC and EM. In terms of the accuracy of the estimates of model parameters, the proposed SMC method compares favorably with other methods.

The remainder of this paper is organized as follows. In Section 2, we describe the system model and problem formulation. In Section 3, we validate the proposed algorithm with simulated data, as well as real data obtained from solid tumors across three major cancer types: prostate adenocarcinoma (PRAD), breast invasive ductal carcinoma (IDC) and lung adenocarcinoma (LUAD). Finally, Section 4 concludes the paper.

Notation-wise in this paper, we denote a vector and a matrix by boldface lower and upper case letters, respectively; *p*(⋅) and *p*(⋅|⋅) denote a probability density function (PDF) and a conditional PDF, respectively; *P*(⋅) and *P*(⋅|⋅) denote a probability and conditional probability mass function, respectively; $N(\mu ,\sigma^{2})$ denotes a Gaussian distribution with mean *μ* and standard deviation *σ*; Binomial(*n*, *p*) denotes a binomial distribution having *n* number of trials and *p* probability of success; Poisson(λ) denotes a Poisson distribution with mean parameter λ; Gamma(*a*_0_, *b*_0_) denotes a gamma distribution with shape parameter *a*_0_ and rate parameter *b*_0_; Beta(*a*_1_, *b*_1_) denotes a beta distribution with shape parameters *a*_1_ and *b*_1_ and Dirichlet(***α***) denotes a Dirichlet distribution with a vector of concentration parameters ***α***.

## System model and problem formulation

### System model

In our model, we assume that a tumor is heterogeneous i.e., it consists of multiple sub-populations, referred to as subclones. Each subclone is assumed to have a unique genotype and at each characterizing mutation locus, we assume that one of the following is the case: (i) none of the alleles is mutated (homozygous wild-type), designated with genotype 0, (ii) one of the alleles is mutated, designated with genotype 0.5, and (iii) both alleles are mutated, designated with genotype 1. Our goal is to estimate the number of subclones, genotypes of all the subclones, and the proportion of each subclone in the tumor samples. To do this, we assume an availability of DNA sequencing data designed to probe tumor heterogeneity. This dataset comes in form of two matrices **Y** and **V** of equal dimension *T* × *S*. *T* and *S* denote the numbers of loci and tumor samples, respectively, and the elements of the two input matrices, *y*_*ts*_ and *v*_*ts*_, *t* = 1, …, *T*, *s* = 1, …, *S*, denote the number of reads that bear a variant sequence and the total number of reads, respectively. We model the matrix of variant counts as follows:

$$\begin{array}{c} y_{ts}\overset{\text{ind.}}{∼}\text{Binomial}\left(v_{ts},p_{ts}\right),\;t=1,\ldots ,T,\;s=1,\ldots ,S, \end{array}$$ (1)

where *p*_*ts*_ is the success probability of obtaining *y*_*ts*_ reads from the total reads *v*_*ts*_ at locus *t* in sample *s*, *t* = 1, …, *T*, *s* = 1, …, *S*. *p*_*ts*_ is interpreted as the weighted sum of the genotypes of all the subclones present in sample *s* as follows:

$$\begin{array}{c} p_{ts}=w_{0s}p+\sum_{c=1}^{C}z_{tc}w_{cs}, \end{array}$$ (2)

where *C* denotes the unknown number of distinct subclones in the tumor samples, *z*_*tc*_ ∈ {0, 0.5, 1} denotes the possible three states for the allelic genotypes at locus *t* in subclone *c* and *w*_*cs*_ denotes the proportion of subclone *c* in tumor sample *s*. In addition, the first term in (2) accounts for experimental and data processing noise, where *p* denotes the relative frequency of variant reads produced as error from upstream data processing and usually takes a small value, close to zero; *w*_0*s*_ absorbs the noise left unaccounted for by {*w*_1*s*_, …, *w*_*Cs*_} [20].

In (2), for all the genomic loci, we arrange the genotypes of all subclones in a *T* × *C* ternary matrix **Z** and we refer to this as the matrix of genotypes. Similarly, we arrange all the *p*’s in a *T*-dimensional column vector **p**, and arrange the respective proportions *w*_0*s*_ and *w*_*cs*_, for all samples, in a *C*′ × *S* matrix **W** and refer to this as the matrix of proportions, where each column of the proportion matrix sums to unity, and *C*′ = *C* + 1. Then (2) can be expressed as a matrix factorization problem, such that:

$$\begin{array}{c} P_{ts}=Z^{\prime }\cdot W, \end{array}$$ (3)

where *p*_*ts*_, an element of **P**_*ts*_, denotes the expected VAF at locus *t* in sample *s* and **Z**′ = [**p Z**]. Given the input read count data, we next describe the proposed SMC algorithm to perform a joint inference on the number of distinct subclones in the tumor samples, the genotype of each subclone and the proportion of each genotype in the tumor samples.

**Algorithm 1** Sample *P*(**z**_*t*_|**Z**_*t*−1_, *α*, *β*) using the categorical Indian buffet process (cIBP)

1: **Z** ← **Z**_*t*−1_

2: β* = 2*β*

3: **if** *t* = 1 **then**

4:  Sample $C_{t}^{new}∼\text{Poisson}\left(\alpha \right)$

5:  **for** $c=1,\ldots ,C_{t}^{new}$ **do**

6:   $z_{tc}\leftarrow \{\begin{array}{cc} 0.5, & \text{with}\;\text{probability}\;(\frac{\beta }{\beta^{*}})\\ 1, & \text{with}\;\text{probability}\;(\frac{\beta }{\beta^{*}}) \end{array}$

7:  **end for**

8: **else**

9:  *C*_+_ ← Number of non-zero columns in **Z**

10:  **for** *c* = 1, …, *C*_+_ **do**

11:   $m_{c1}\leftarrow \sum_{r=1}^{t-1}I\left(z_{rc}=0.5\right)$

12:   $m_{c2}\leftarrow \sum_{r=1}^{t-1}I\left(z_{rc}=1\right)$

13:   *m*_*c*_ ← *m*_*c*1_ + *m*_*c*2_

14:   $z_{tc}\leftarrow \{\begin{array}{cc} 0, & \text{with}\;\text{probability}\;[1-\frac{m_{c}}{t}]\\ 0.5, & \text{with}\;\text{probability}\;[(\frac{m_{c}}{t})\times (\frac{\beta +m_{c1}}{\beta^{*}+m_{c}})]\\ 1, & \text{with}\;\text{probability}\;[(\frac{m_{c}}{t})\times (\frac{\beta +m_{c2}}{\beta^{*}+m_{c}})] \end{array}$

15:  **end for**

16:  Sample $C_{t}^{new}∼\text{Poisson}(\frac{\alpha }{t})$

17:  **for** $d=\left(C_{+}+1\right),\ldots ,\left(C_{+}+C_{t}^{new}\right)$ **do**

18:   $z_{td}\leftarrow \{\begin{array}{cc} 0.5, & \text{with}\;\text{probability}\;(\frac{\beta }{\beta^{*}})\\ 1, & \text{with}\;\text{probability}\;(\frac{\beta }{\beta^{*}}) \end{array}$

19:  **end for**

20: **end if**

### State-space formulation

In this section, we succinctly describe our state-space formulation of the deconvolution problem we set up in (3) with the details described in S1 File. At time step *t*, we consider the *t*^*th*^ row of the input read count matrices, as the observation at that particular time. Subsequently, because we are interested in constructing the ternary genotype matrix **Z** (with an unknown number of columns) sequentially, one row after the other, using the cIBP (details in the S1 File), we consider the *t*^*th*^ row of the genotype matrix as the hidden state at *time t*, and then, the proportion of the subclones in the tumor samples, matrix **W** and *p* are considered as the parameters of our state-space model. Thus, the state transition equation is stated as follows:

$$\begin{array}{c} P(z_{t}|Z_{t-1},\alpha ,\beta ), \end{array}$$ (4)

where **Z**_*t*−1_ denotes the previous *t* − 1 rows in the genotype matrix **Z**, *α* and *β* are constants, to be supplied by the user. The reasonable range for both constants are discussed in S1 File and the algorithm to sample from (4) is presented in **Algorithm 1** as follows.

The genotype matrix at time step *t*, **Z**_*t*_ is implicitly constructed from the genotype matrix in the previous time step *t* − 1, **Z**_*t*−1_. In the construction process, if new non-zero column(s) is/are introduced in **Z**_*t*_, then the subclone proportion matrix **W** would be augmented with an equivalent number of rows. Thus, **W** requires some re-parameterization to account for such change in dimension. Specifically, we rewrite $w_{cs}=\theta_{cs}/\sum_{c^{\prime }=0}^{C}\theta_{c^{\prime }s}$. This implies that instead of estimating *w*_*cs*_ directly, we estimate *θ*_*cs*_, and then obtain *w*_*cs*_ from the estimates of *θ*_*cs*_. Such re-parameterization ensures that each column of **W** sums to unity at every time step.

Moreover, since we are interested in the final estimates of the model parameters **W** and *p*, we create artificial dynamics for these parameters using the random walk model as follows:

$$\begin{array}{c} \begin{array}{c} \phi_{t}∼p\left(\phi_{t}|\phi_{t-1}\right)=N\left(\phi_{t-1},\sigma^{2}\right),\\ \phi_{t}\in \left\{p,\theta_{cs},c=0,1,\ldots ,C,s=1,\ldots ,S\right\}, \end{array} \end{array}$$ (5)

where *σ* denotes the standard deviation. Hence, (4) and (5) fully describe the system state transition. Similarly, the observation at time *t* is given by:

$$\begin{array}{c} \begin{array}{cc} y_{t}∼P\left(y_{t}|Z_{1:t},W,p\right) & =P(y_{t}|z_{t},W,p)\\ & =\prod_{s=1}^{S}\text{Binomial}\left(y_{ts}|v_{ts},p_{ts}\right), \end{array} \end{array}$$ (6)

where **y**_*t*_ denotes the observation at time *t* (which is conditionally independent of the previous observations **Y**_*t*−1_ given the state **z**_*t*_), i.e., the *t*^*th*^ row of **Y**. (6) describes the measurement model for the system. Finally, (4)–(6) completely describe our proposed state-space model for estimating the number, genotypes and proportions of subclones in tumor samples.

**Algorithm 2** SMC algorithm for inferring subclonal structure

**Input**: **Y**, **V**.

1: Initialize *N* particles ${\left\{z_{0}^{i},p_{0}^{i},W_{0}^{i}\right\}}_{i=1}^{N}$

2: **for** *t* = 1, …, *T* **do**

3:  **for** *i* = 1, …, *N* **do**

4:   Sample $z_{t}^{i}$ from $Z_{t-1}^{i}$ using **Algorithm 1**.

5:   *n*_1_ ← number of columns in $Z_{t-1}^{i}$

6:   *n*_2_ ← length of $z_{t}^{i}$

7:   *d* ← (*n*_2_ − *n*_1_)

8:   **if** *d* = 0 **then**

9:

$$Z_{t}^{i}\leftarrow [\begin{array}{c} Z_{t-1}^{i}\\ z_{t}^{i} \end{array}]$$

10:    Sample $W_{t}^{i}$ using (5)

11:   **else**

12:

$$Z_{t}^{i}\leftarrow [\begin{array}{c} Z_{t-1}^{i}\;0\\ z_{t}^{i} \end{array}]$$

13:    Sample $W_{t}^{i}$ using (5).

14:    Sample new rows of $W_{t}^{i}$ from the prior in (9).

15:   **end if**

16:   Calculate ${\tilde{w}}_{t}^{i}$ using (8)

17:  **end for**

18:  Normalize the weights

19:  Perform resampling

20: **end for**

21: Final particles of the genotype matrix (${\left\{Z_{T}\right\}}_{i=1}^{N}$) and proportion matrix (${\left\{W_{T}\right\}}_{i=1}^{N}$) consist of varying number of columns and rows, respectively. Estimate of the number of subclones is obtained from the number of columns of the genotype particles (equivalently the number of rows of the proportion particles). Details of how the posterior estimates of all the unknown variables are obtained from the final particles and weights, using the procedures highlighted in [20, 27], are discussed in S1 File.

### The SMC algorithm

We summarize the SMC filtering framework employed to make inference about the number of subclones, genotype of each subclone and the proportion of each subclone in the tumor samples, which are the states and the parameters of our proposed state-space model. Details of our proposed algorithm are presented in S1 File.

Consider the general dynamic system with hidden state variable **x**_*t*_, in our case consisting of categorical variables **z**_*t*_ and continuous variables *φ*_*t*_, $\phi_{t}\in \left\{p_{0}^{t},\theta_{cs}^{t},c=0,1,\ldots ,C,s=1,\ldots ,S\right\}$, and measurement variable **y**_*t*_, where there is an initial state model *p*(**x**_0_), and ∀*t* ≥ 1, a state transition model given in (4) and (5) and an observation model given in (6). The sequence **X**_*t*_ = {**x**_1_, **x**_2_, …, **x**_*t*_} is not observed and we want to estimate it for each time step, given that we have the observations **Y**_*t*_ = {**y**_1_, **y**_2_, …, **y**_*t*_}. Our goal is to approximate the posterior distribution of states *p*(**X**_*t*_|**Y**_*t*_) using samples drawn from it. Getting such samples from *p*(**X**_*t*_|**Y**_*t*_) is not feasible, at least in our model. However, we can still implement an estimate using *N* samples (particles), ${\left\{X_{t}^{i}\right\}}_{i=1}^{N}$, taken from another distribution, *q*(**X**_*t*_|**Y**_*t*_), whose support includes the support of *p*(**X**_*t*_|**Y**_*t*_) (importance sampling theorem), and each particle is accompanied by a weight *w*_*i*_ such that $\sum_{i=1}^{N}w_{i}=1$ (see S1 File for detail). Thus, the pair ${\left\{X_{t}^{i},w_{1:t}^{i}\right\}}_{i=1}^{N}$ is said to be properly weighted with respect to the distribution *p*(**X**_*t*_|**Y**_*t*_), and the approximation $\hat{p}\left(X_{t}|Y_{t}\right)$ is then given by:

$$\begin{array}{c} \begin{array}{c} \hat{p}\left(X_{t}|Y_{t}\right)=\sum_{i=1}^{N}w_{t}^{i}\delta \left(X_{t}-X_{t}^{i}\right),\;\text{where}\;\delta \left(u\right)=\{\begin{array}{cc} 1, & \text{if}\;u=\underline{0}\\ 0, & \text{otherwise}. \end{array} \end{array} \end{array}$$ (7)

Next, the importance sampling theory is generalized to obtain a sequential algorithm as follows. We assume that, at time step *t* − 1, we have already drawn the weighted particles ${\left\{X_{t-1}^{i},w_{t-1}^{i}\right\}}_{i=1}^{N}$ from the importance distribution *q*(**X**_*t*−1_|**Y**_*t*−1_) to approximate the target posterior distribution *p*(**X**_*t*−1_|**Y**_*t*−1_). At time step *t*, we can now draw particles ${\left\{X_{t}^{i}\right\}}_{i=1}^{N}$ from the importance distribution *q*(**X**_*t*_|**Y**_*t*_) as follows: (i) draw new state particles for the time step *t* as $x_{t}^{i}∼p\left(x_{t}|X_{t-1}^{i}\right)$ from (4) and (5), and (ii) write ${\left\{X_{t}^{i}\right\}}_{i=1}^{N}={\left\{x_{t}^{i},X_{t-1}^{i}\right\}}_{i=1}^{N}$. Then, the unnormalized weights at time step *t* are obtained from the normalized weights at time step *t* − 1 and the measurement model in (6) as follows:

$$\begin{array}{c} \begin{array}{cc} {\tilde{w}}_{t}^{i} & \propto w_{t-1}^{i}p\left(y_{t}|x_{t}^{i}\right)\\ & =w_{t-1}^{i}p\left(y_{t}|z_{t}^{i},W_{t}^{i}\right), \end{array} \end{array}$$ (8)

and the unnormalized weights ${\tilde{w}}_{t}^{i}$ are normalized to sum to unity. However, since the variance of the weights increases over time, we perform resampling at every time step, owing to the choice of our importance distribution (see S1 File for detail) [28–31], discarding the ineffective particles and multiplying the effective ones. The resampling procedure [25] is briefly summarized as follows:

- Interpret each weight $w_{t}^{i}$ as the probability of obtaining the particle index *i*.
- Draw *N* particles from the discrete probability distribution $\left\{w_{t}^{i}\right\}$ and replace the old particle set with this new one.
- Set all weights to the constant value $w_{t}^{i}=1/N$.

Finally, the proposed SMC algorithm for estimating the states and the parameters of our state-space model is presented in **Algorithm 2**. The algorithm is initialized by taking samples from the prior distributions of the parameters. We assume the following:

$$\begin{array}{l} \theta_{0s}\overset{i.i.d}{∼}\text{Gamma}\left(a_{0},1\right),\;s=1,\ldots ,S,\\ \theta_{cs}\overset{i.i.d}{∼}\text{Gamma}\left(a_{1},1\right),\;s=1,\ldots ,S,c=1,\ldots ,C,\;\text{and}\\ \;\;p∼\text{Beta}\left(a_{00},b_{00}\right), \end{array}$$ (9)

such that $w_{cs}=\theta_{cs}/\sum_{c^{{}^{\prime }}=0}^{C}\theta_{c^{{}^{\prime }}s}$ and consequently, $\sum_{c^{{}^{\prime }}=0}^{C}w_{c^{{}^{\prime }}s}=1$. We report the posterior estimates of all the unknown variables using the procedure highlighted in [27], with the details discussed in S1 File.

## Results

### Application to simulated datasets

To validate our method, we generated multiple simulated datasets for different combinations of the number of subclones *C*, average sequencing depth *r*, sample size *S* and the number of loci *T*. Specifically, we considered *C* ∈ {3, 4, 5} subclones, *S* ∈ {3, 4, …, 15} tumor samples, we fixed the average sequencing depth *r* = 100 and also the number of loci, *T* = 20. For each combination of the number of subclones, sample size, average sequencing depth and number of loci, we produced 10 datasets as follows: (i) the total read count at locus *t* in sample *s*, i.e., *v*_*ts*_ is generated from Poisson(*r*), (ii) each column of the proportion matrix is independently generated from Dirichlet([*a*_0_, *a*_1_, …, *a*_*C*_]), *a*_0_ = 0.1 and *a*_*c*_; *c* ∈ {1; …, *C*} is randomly chosen from the set {2, 4, 5, 6, 7, 8}, (iii) each entry of the genotype matrix is independently generated from Discrete([0.5 0.1 0.4]) and set *p* = 0.02, (iv) the success probability *p*_*ts*_ is computed following (2), and then, (v) *y*_*ts*_, the variant count, is generated as an independent sample from Binomial(*v*_*ts*_, *p*_*ts*_).

To quantify the performance of the proposed algorithm, we define the following metrics: genotype reconstruction error (*e*_*Z*_), proportion error (*e*_*W*_) and the error of the success probabilities ($e_{p_{ts}}$) as follows:

$$\begin{array}{c} \begin{array}{c} e_{Z}=\frac{1}{TC}\sum_{t=1}^{T}\sum_{c=1}^{C}|{\hat{z}}_{tc}-z_{tc}|,\;e_{W}=\frac{1}{CS}\sum_{c=0}^{C}\sum_{s=1}^{S}\left|{\hat{w}}_{cs}-w_{cs}\right|, \end{array} \end{array}$$

$$\begin{array}{c} \begin{array}{c} e_{p_{ts}}=\frac{1}{TS}\sum_{t=1}^{T}\sum_{s=1}^{S}\left|{\hat{p}}_{ts}-p_{ts}\right|,\;\text{where}\;{\hat{p}}_{ts}=\hat{p}{\hat{w}}_{0s}+\sum_{c=1}^{C}{\hat{z}}_{tc}{\hat{w}}_{cs}. \end{array} \end{array}$$

However, because this is a blind decomposition, it is not clear a priori which column of the estimated genotype matrix $\hat{Z}$ corresponds to which column of the true genotype matrix **Z**. We resolve this by calculating *e*_*Z*_ with every permutation of the columns of $\hat{Z}$ and then select the permutation that results in the smallest value. The selected permutation is then used in computing *e*_*W*_ and $e_{p_{ts}}$.

**Fig 1:**
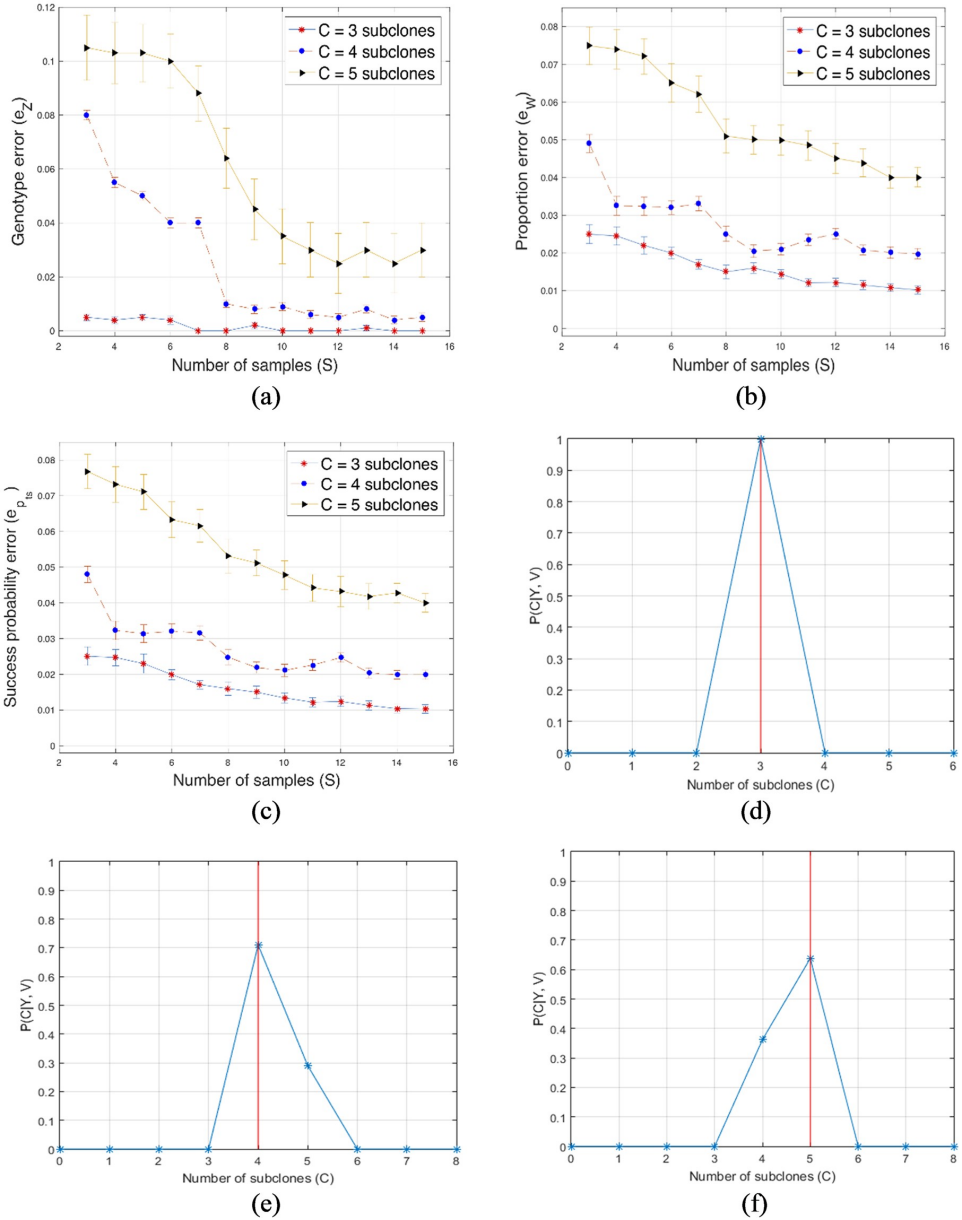
Simulation results for the proposed algorithm. (a), (b) and (c): Plots of the genotype error (*e*_*Z*_), proportion error (*e*_*W*_) and error of success probability ($e_{p_{ts}}$) versus different sample sizes for subclones *C* ∈ {3, 4, 5}. (d), (e) and (f): Posterior distributions of *C*, for *C* = 3, 4, and 5.

For every combination of the number of subclones, sample size, average sequencing depth and number of loci, we computed the average and the standard deviation of the genotype error, proportion error and the error of the success probabilities over the 10 datasets in each group. The results are presented in Fig 1(a)–1(c) where the standard deviation is the vertical line above and below the average value in the errorbar plots. These results show that the performance of the proposed algorithm improves with an increase in the number of tumor samples. Also, when the number of subclones in the samples is minimal, estimation of model parameters becomes more accurate. For *T* = 20, *r* = 100, *S* = 10 and *C* ∈ {3, 4, 5}, we present, in Fig 1(d)–1(f), the estimated posterior distributions of *C*. In the three cases, the maximum a posteriori (MAP) estimates of *C* (marked with red vertical lines) are 3, 4 and 5. It should be noted that in the implementation of the proposed algorithm, the estimates of other model parameters are conditional on the MAP estimate of *C*. This is discussed further in S1 File.

**Fig 2:**
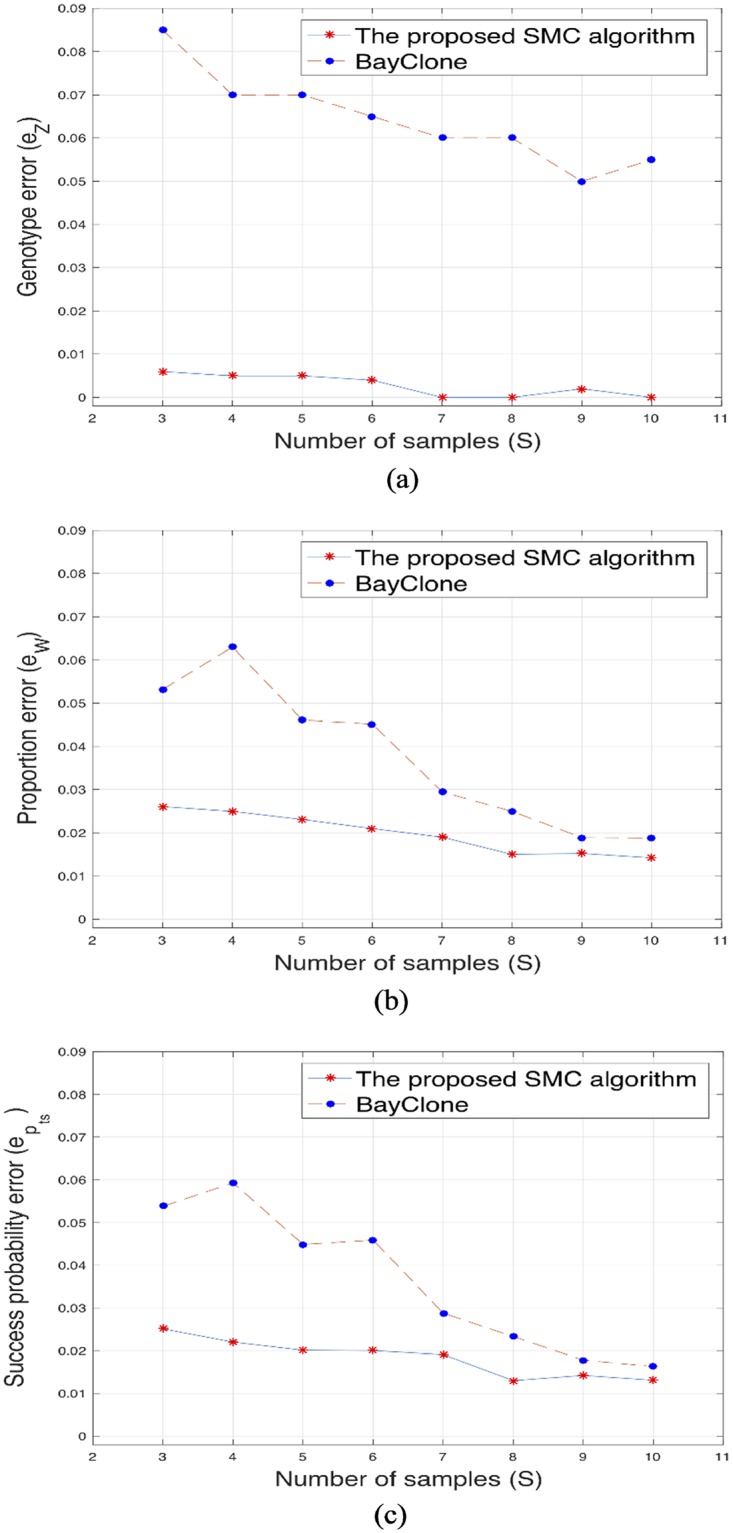
The proposed algorithm and BayClone. (a), (b) and (c): Plots of the genotype error (*e*_*Z*_), proportion error (*e*_*W*_) and error of success probability ($e_{p_{ts}}$) versus different sample sizes for the proposed algorithm and BayClone.

**Fig 3:**
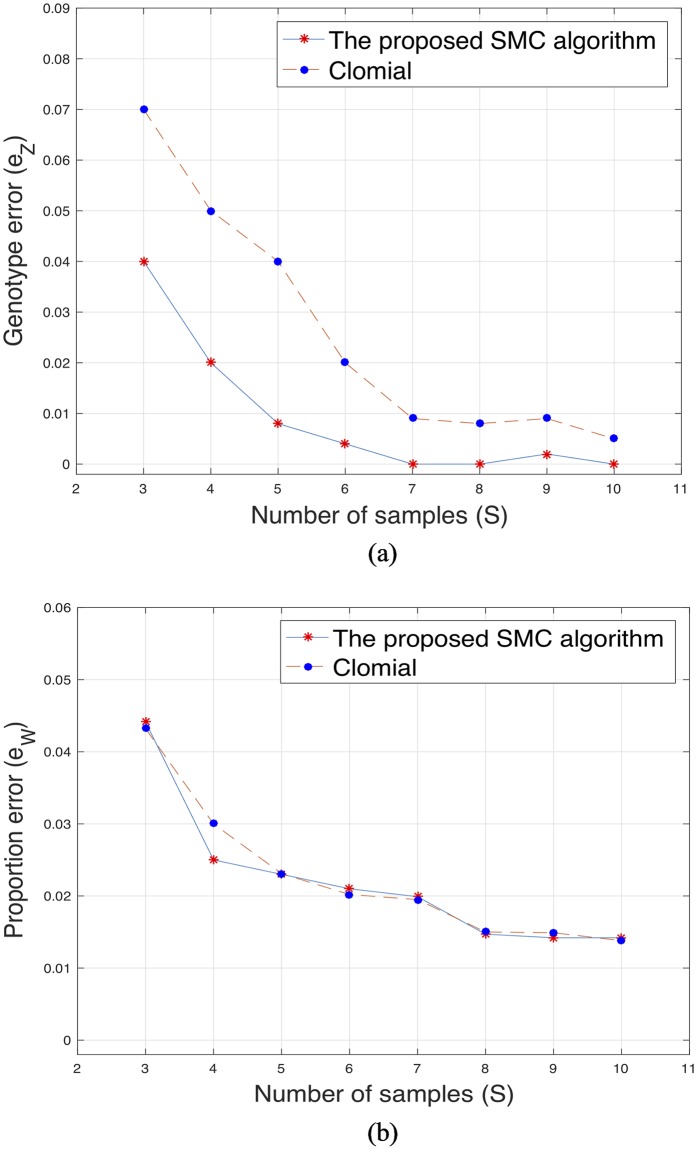
The proposed algorithm and Clomial. (a) and (b): Plots of the genotype error (*e*_*Z*_) and proportion error (*e*_*W*_) versus different sample sizes for the proposed algorithm and Clomial.

Further, we compared our proposed algorithm with BayClone [20], an algorithm with similar model assumption and also with Clomial [13]. For the comparison with Clomial, the true genotype matrix only includes two categories i.e. 0 for an absence of mutation and 0.5 for the presence of mutation and each entry of the matrix is generated from Discrete([0.3 0.7]). In computing the errors for Clomial, we viewed a 1 in the estimated genotype matrix as 0.5 for consistency with the true matrix. The results of the simulated data for three subclones, different sample size, average sequencing depth of 100 and 50 loci are presented in Figs 2 and 3. Fig 3 does not include the error of success probability because Clomial only estimates the genotype and the proportion matrices. The runtime for the proposed algorithm, BayClone and Clomial for sample size *S* = 5, number of subclones *C* = 3, average sequencing depth *r* = 100 and 50 loci are 782, 1454 and 768 seconds, respectively, on a 3.5 GHz Intel 8 cores running MATLAB. Lastly, we investigated the performance of the algorithms when the number of loci is very large since this is often a source of computational issue in some of the existing methods [19]. The result for 2000 and 5000 genomic loci are presented in Table 1 (the results for 2000 and 5000 loci are with and without brackets, respectively). For the proposed algorithm, we noticed a slight improvement in the estimate of the proportion when the number of loci is large. In the case of the two other algorithms, we observed a slight increase in the genotype and proportion errors with large genomic loci.

**Table 1:** Comparison of algorithms on large datasets.

	Genotype error	Proportion error	Runtimes (seconds)
Proposed algorithm	0.0040 [0.0050]	0.0121 [0.0116]	2.754e4 [5.707e4]
BayClone	0.1000 [0.0950]	0.0632 [0.0724]	5.032e4 [1.363e5]
Clomial	0.0850 [0.0500]	0.0548 [0.0550]	2.736e4 [5.688e4]

### Application to solid tumor datasets

#### Data pre-processing

The somatic mutation data of real solid tumors come from the American Association for Cancer Research (AACR) Genomics Evidence Neoplasia Information Exchange (GENIE) project [1]: Version 2.0.0, which are accessible on the Sage Synapse platform (with Synapse ID: syn11310744) [32]. We performed three filtering criteria before creating the final data set to run our algorithm. (i) The data release includes genomic records collected by eight participating institutions. To control the batch effect, we selected samples from Memorial Sloan Kettering (MSK) Cancer Center given the fact that they provide matched tumor-normal (rather than tumor-only) sequence data and their sample size is the largest. (ii) We selected patients who have at least three samples with somatic mutation data. (iii) We further filtered out samples so that the remaining data contain information for at least three patients for each cancer type. As a result, the data set we retained include 36 samples (of 10 patients) with prostate adenocarcinoma (PRAD), 18 samples (of 6 patients) with breast invasive ductal carcinoma (IDC) and 9 samples (of 3 patients) with lung adenocarcinoma (LUAD).

To create the input count matrices for the proposed algorithm, we combined count data of all the samples from the same patient by the union of their mutated gene symbols. Regarding the entries for which the mutation of the corresponding gene was not detected in some samples, we imputed the values with the average counts of the matched normal samples. For instance, we assume that there are three samples (A, B, C) from a specific patient and samples A and B have mutations at gene G while sample C does not. In the combined total (or alteration) count matrix of this patient, we used the average of total (or alteration) counts for gene G of the matched normal samples of A and B to be the imputed count of C for gene G in the combined matrices.

#### Inferred subclonal structure and phylogenetic trees

We illustrated the use of our algorithm on the three solid cancer types: PRAD, IDC and LUAD. We applied our algorithm on the data of every patient, resulting in the inferred subclonal landscape, which contains the information of the genotypes, the proportions of each subclone as well as the possible phylogenetic tree. Some of the model parameter estimates are presented and the others, including the posterior distributions of the number of subclones, are in S1 Tables and S1 Figs.

A phylogenetic tree depicts the evolutionary history of cancer progression. Based on the inferred subclonal genotypes, drawing insight from the approach in [13], we manually constructed a phylogenetic tree for each patient, in which the root is always the normal subclone, each node represents a subclonal population, and the mutations that occurred between the parent and the offspring nodes are shown on the edges. Moreover, since our algorithm is able to identify both heterozygous and homozygous mutations, we annotated those mutations which were inferred as homozygous. We reasoned that investigating the subclonal results combined with the phylogenetic characteristics has the potential to provide evidence for the validity of our method.

#### Driver mutations found on edges connected to the root of the phylogenetic trees

We observed that genes with well known driver mutations for one cancer type are located on the edges that are connected to the root of the phylogenetic tree of patients with that cancer. This is consistent with the fact that are somatic mutations in a gene that confer a selective advantage on cancer cells, which are believed to be involved in cancer initiation and clonal expansions [33].

**Fig 4:**
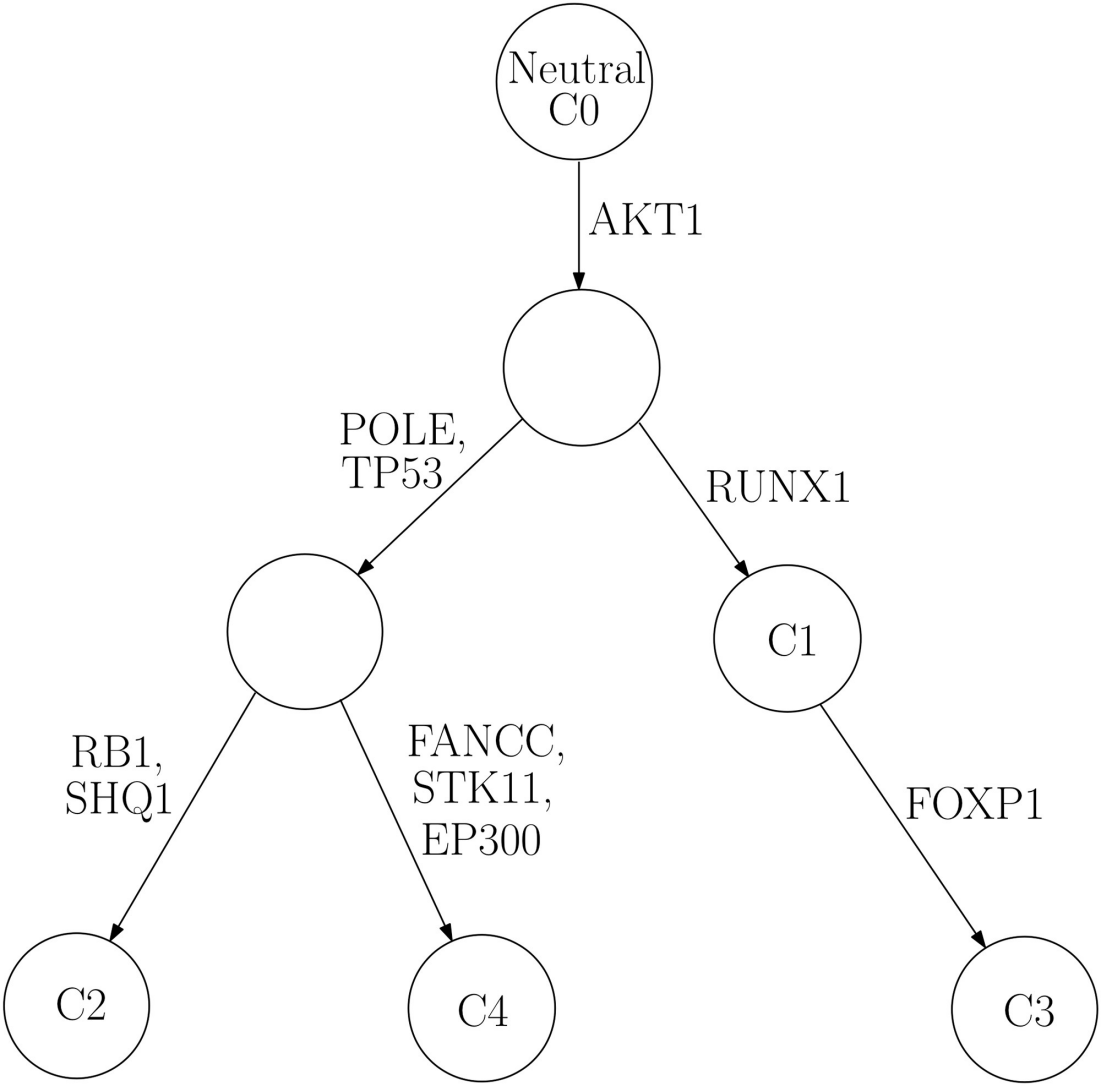
Phylogenetic tree for IDC_0000525. Constructed phylogenetic tree for patient IDC_0000525.

**Fig 5:**
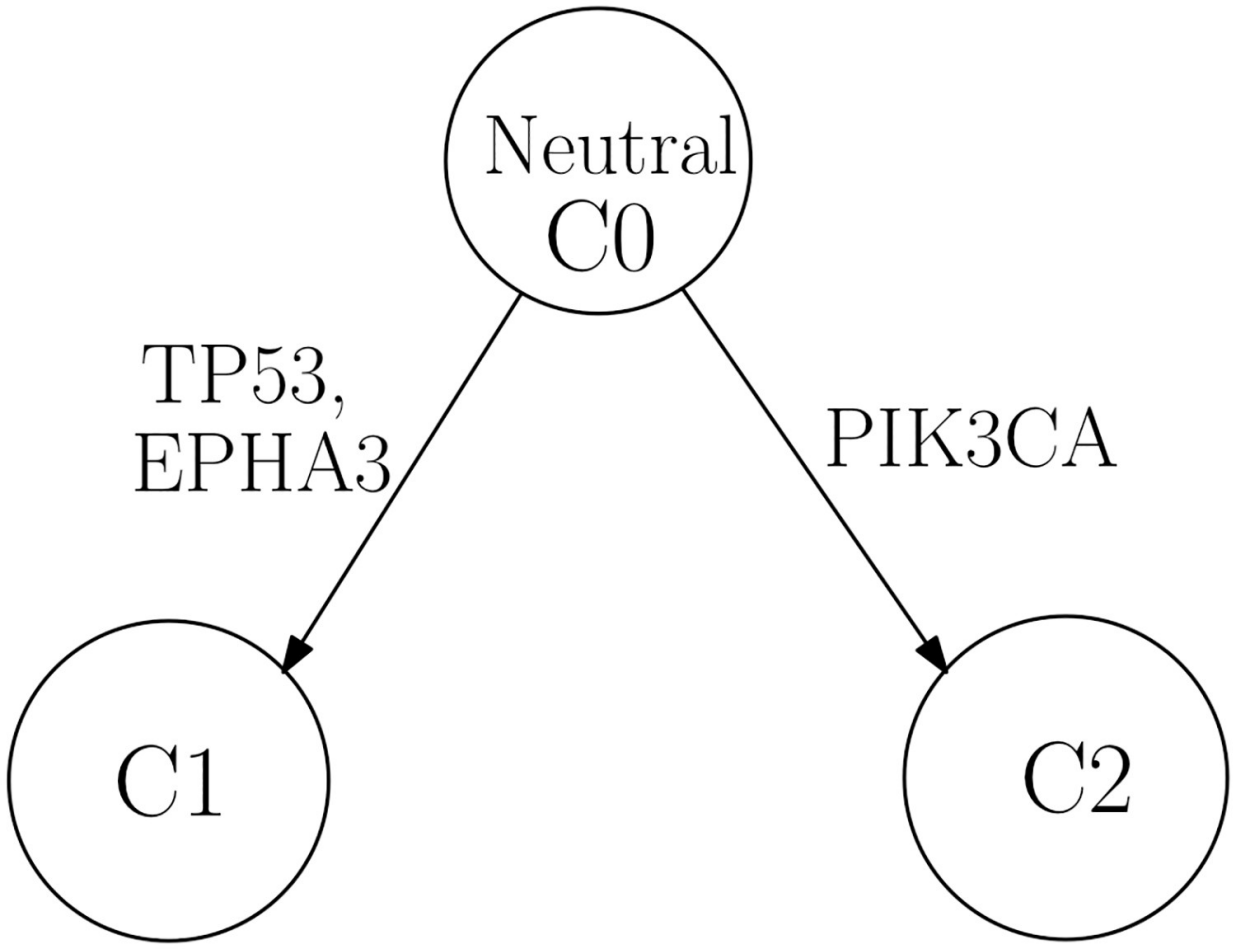
Phylogenetic tree for IDC_0000690. Constructed phylogenetic tree for patient IDC_0000690.

Specifically, in each of the six instances of IDC, we found that either gene PIK3CA or gene AKT1 is placed on the edge directly connected to the neutral/normal subclone. Two examples are shown in Fig 4 (IDC_0000525) and Fig 5 (IDC_0000690) and the corresponding estimated genotype matrices are shown in Tables 2 and 3, respectively. The inferred results for other IDC patients can be found in S1 Figs and S1 Tables. Somatic mutations occurring in oncogenes PIK3CA and AKT1 have been widely reported in breast cancer [34–36]. PIK3CA is the most frequently mutated gene found in breast cancer [37], and it is an integral component of the phosphatidylinositol 3 kinase (PI3K) signaling pathway. AKT1, one of the three isoforms of the protein kinase AKT, is also a mediator in the downstream of the PI3K pathway and it plays a key role in promoting cell survival by inhibiting apoptosis. Its over-activation has been implicated in tumorigenesis [35–38]. The dysregulation of the PI3K/AKT pathway has been demonstrated in different solid tumors including breast cancer, and it has been suggested that this dysregulation is associated with the increased mutations in pathway genes PIK3CA and AKT1 [36, 39].

**Table 2:** Estimated genotype for IDC_0000525.

Gene name	C1	C2	C3	C4
TP53	0	0.5	0	0.5
AKT1	0.5	0.5	0.5	0.5
RUNX1	0.5	0	0.5	0
POLE	0	0.5	0	0.5
FANCC	0	0	0	0.5
STK11	0	0	0	0.5
EP300	0	0	0	0.5
RB1	0	0.5	0	0
FOXP1	0.5	0	0.5	0
SHQ1	0	0.5	0	0

**Table 3:** Estimated genotype for IDC_0000690.

Gene name	C1	C2
TP53	0.5	0
PIK3CA	0	0.5
EPHA3	0.5	0

**Fig 6:**
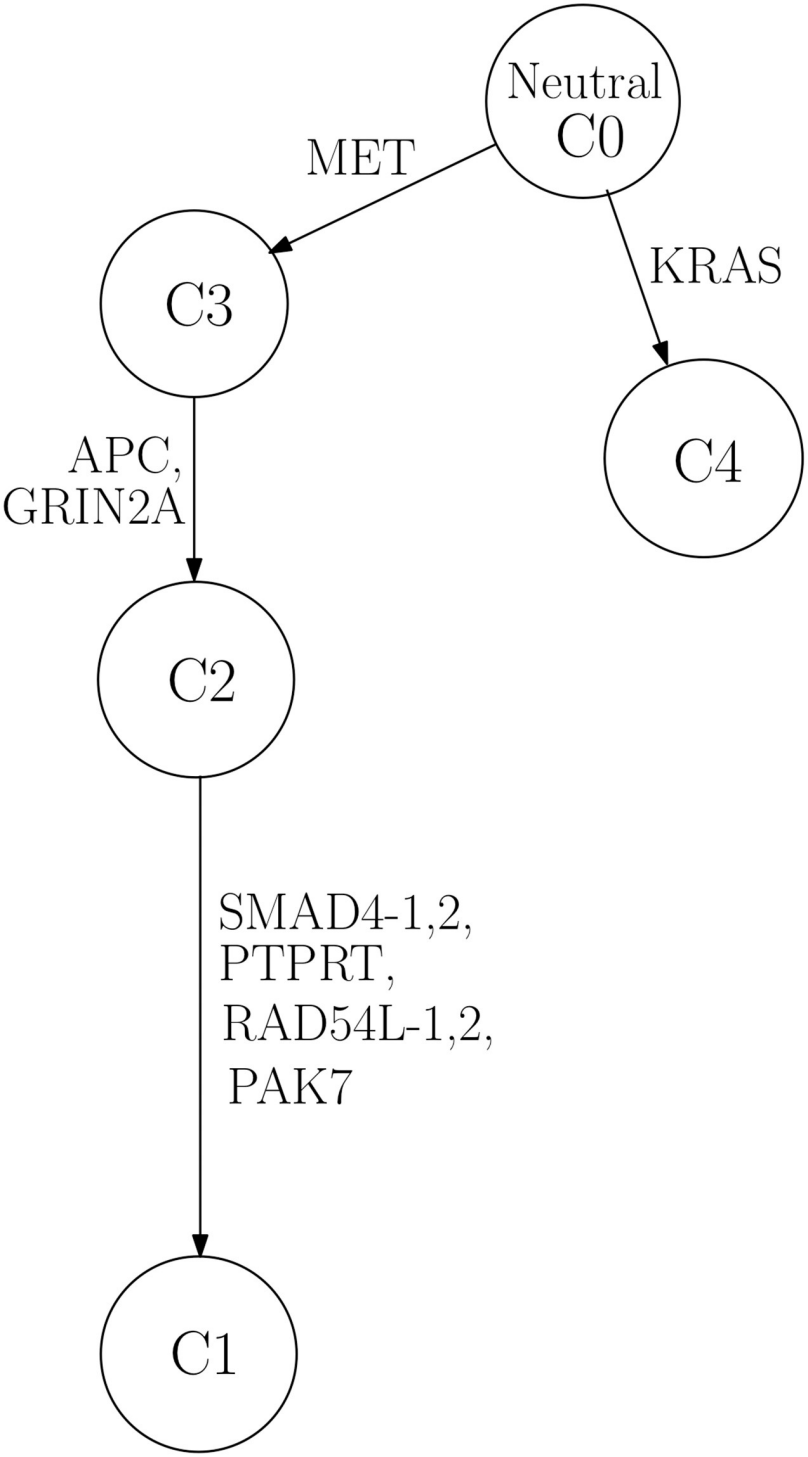
Phylogenetic tree for LUAD_0000978. Constructed phylogenetic tree for patient LUAD_0000978.

In the case of LUAD, KRAS and EGFR have mutations found prevalent in patients [40–42]. Despite the small number of patients, the constructed phylogenetic trees showed consistent results. First, among the three LUAD patients, two of them harbor somatically mutant KRAS and the remaining one has mutation in EGFR, which also reflects the well-known mutual exclusiveness of these two driver mutations [43]. Fig 6 and Table 4 display the case of patient LUAD_0000978, from which we can find that KRAS is marked on the edge connected to the root in the phylogenetic tree, indicating its oncogenic role. A previous study analyzing somatic mutation data of non-small cell lung cancer by a different method also found that KRAS and EGFR mutations were present in the founder clone in their results, suggesting that it is likely that these mutations are initiating events for lung cancer [40].

**Table 4:** Estimated genotype for LUAD_0000978.

Gene name	C1	C2	C3	C4
SMAD4	1	0	0	0
PTPRT	0.5	0	0	0
RAD54L	1	0	0	0
APC	0.5	0.5	0	0
GRIN2A	0.5	0.5	0	0
PAK7	0.5	0	0	0
MET	0.5	0.5	0.5	0
KRAS	0	0	0	0.5

#### Genotype assignments validated by the tree structures

**Fig 7:**
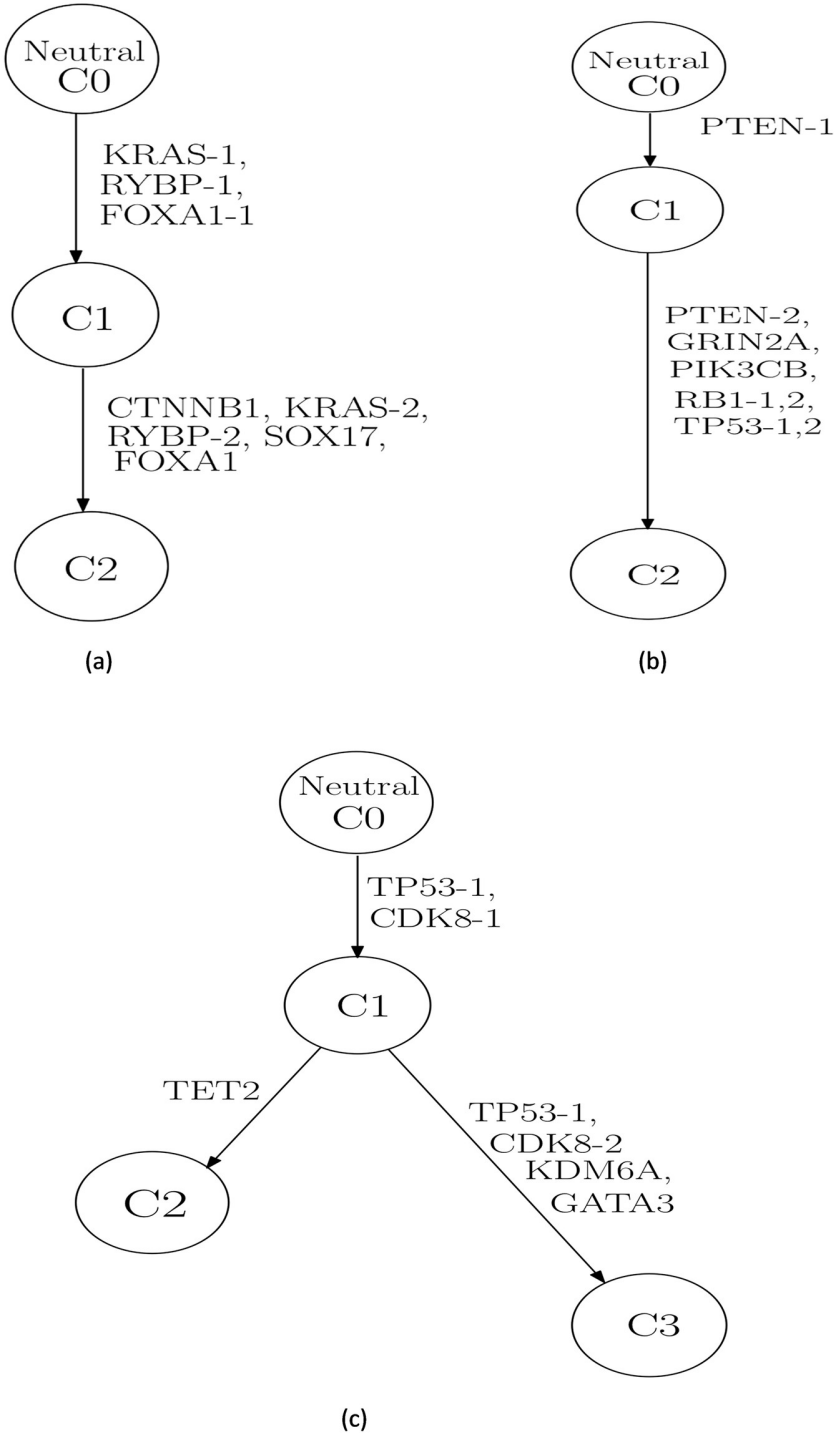
Phylogenetic trees for patients with PRAD. Constructed phylogenetic tree for patients: (a) PRAD_0000655, (b) PRAD_0003101 and (c) PRAD_0003511.

One of the advantages of the proposed algorithm is that for each gene, it can consider three different categories of genotype: wild-type, heterozygous and homozygous. This feature was validated by analyzing the hierarchical structure of the inferred phylogenetic trees. Given that one of our assumptions is that a mutation never disappears in the entire phylogeny, if a mutant gene were assigned different genotypes in different subclones, the subclone(s) with homozygous mutations should be descendant(s) of the subclone(s) with heterozygous mutations. This implies that the paternal and the maternal alleles (or vice versa) of this gene became mutated consecutively, along the clonal evolution. Such situations apply to three cases of PRAD patients: PRAD_0000655, PRAD_0003101, PRAD_0003511 (Fig 7), constructed from the inferred genotype matrices in S1 Tables. For example, in patient with ID “PRAD_0003101”, the inferred decomposition results in S1 Tables showed that there are two subclones (referred to as subclone 1 and subclone 2, respectively) in addition to the normal one. Both subclone 1 and subclone 2 harbor mutations in gene PTEN; however, the respective genotypes are different: “0.5” (i.e. heterozygous) for subclone 1 while “1” (homozygous) for subclone 2. The constructed phylogenetic tree revealed concordant result (Fig 7(b)) that subclone 2 is the offspring node of subclone 1, suggesting that an additional mutation event occurred in PTEN during this clonal expansion which resulted in the change in genotype.

#### Inferred subclonal proportions along the phylogeny indicate tumor progression

Furthermore, the inferred subclonal proportions along with the tree structures provide more evidence to validate our algorithm. For the same patient that we discussed above i.e., “PRAD_0003101”, there are three metastatic samples available among which one was obtained when the patient was 68 years old (referred to as M1) and the other two were obtained when he was 69 years old (referred to as M2 and M3). We found that the proportions of subclone 2 in M2 (96%) and M3 (86%) samples are much higher than the one for M1 sample (29%), and cases for subclone 1 to the contrary S1 Tables. Meanwhile, we also observed similar results for another patient with ID “PRAD_0001204”, who has two primary tumor samples and one metastatic sample (S1 Tables and S1 Figs). In this case, subclone 1 descends from subclone 2, and the highest proportion of subclone 1 can be found in the metastatic sample, which was also obtained when the patient was older. These findings imply that as the patient aged or the cancer metastasized, the mutations specific to the descendant subclone gained cells survival advantage, promoting cell proliferation, and hence resulted in the increasing proportion of the subclone in samples.

## Discussion

The inherent heterogeneity in tumor samples often results in setbacks when cancer patients undergo treatment. The samples consist of different subpopulations of cancerous cells, each characterized by a distinct mutational profile. Inference of these profiles and the proportion of each subpopulation in the samples can improve personalized medicine e.g. preventing cancer relapse and helping in cancer prognosis. We proposed an efficient sequential algorithm for estimating the mutational profile of each cancer cell subpopulation and their respective proportions in the tumor samples. With simulated datasets, we performed experiments to validate our algorithm. We applied our algorithm to real tumor samples, covering three solid cancer types, PRAD, IDC, and LUAD.

By analyzing the inferred genotype landscape results, we found evidence supporting the validity of our method in several ways. For example, many well-known driver mutations specific to cancer types were found in the edges directly connected to the root in the inferred phylogenetic tree. The position of these somatic mutations indicates their roles in cancer initiation and expansion. For example, somatic mutations in genes PIK3CA and AKT1 were identified as driver events for breast cancer, suggesting malfunction of PI3K/AKT pathway in cancer [39]. Such characteristics were consistently observed across different patients included in this study.

We also evaluated our algorithm by investigating the phylogenetic tree structures, which could imply the cancer progression history in patients. The algorithm is able to distinguish the genotype of a mutation among wild-type, heterozygous and homozygous. Consistent with one of our assumptions that a somatic mutation will not disappear, our results revealed that if a mutant gene were assigned different genotypes in different subclones, the subclone(s) with homozygous mutations was always the descendant(s) of the subclone(s) with heterozygous mutations, indicating the order of mutation events on different alleles during the clonal expansion. Moreover, we observed increasing proportions of leaf subclones in more advanced samples than less advanced ones, such as metastatic samples versus primary samples, from the identical patients, suggesting the proliferation of cells in these subclones due to the survival advantages by acquiring more mutations during the cancer progression [1].

Lastly, the proposed algorithm can handle any number of mutations in an accurate and computationally efficient manner.

## Supporting information

S1 Figs Constructed phylogenetic trees from the estimated subclonal genotypes. (PDF)

S1 Tables Estimated genotypes for the subclones. Tables of the estimated genotypes of subclones.
(PDF)

S1 File Details description of the algorithms. Detailed description of the sampling procedure from the prior distribution of a ternary matrix using the cIBP, sequential construction of a ternary matrix, and the detailed description of our proposed SMC algorithm.
(PDF)
