# Supplementary material for: A sequential Monte Carlo algorithm for inference of subclonal structure in cancer
Source: PLoS One. 2019 Jan 25;14(1):e0211213. doi: 10.1371/journal.pone.0211213 (PMC6347199; doi:10.1371/journal.pone.0211213)
Supplement: S1 Figs — (PDF) [file pone.0211213.s001.pdf]

S1 Figures

Paper Title: A sequential Monte Carlo algorithm for inference of subclonal structure in cancer

Authors: Oyetunji Ogundijo, Kaiyi Zhu, Xiaodong Wang and Dimitris Anastassiou

Part A

Here, we present the manually reconstructed phylogenetic trees from the genotype matrices of subclones inferred from the 13 other cancer patients (IDC_0000247, IDC_0002756, IDC_0004183, IDC_0006161, LUAD_0001303, LUAD_0012569, PRAD_0001204, PRAD_0001242, PRAD_0002273, PRAD_0002898, PRAD_0005970, PRAD_0006108 and PRAD_0000377). These results are shown in Figures 1 – 4. The phylogenetic trees obtained from analyzing the dataset from cancer patients IDC_0000247, IDC_0002756, IDC_0004183 and IDC_0006161 are presented in Figure 1. In Figure 2, we present the reconstructed trees for cancer patients LUAD_0001303 and LUAD_0012569. Figure 3 consists of the reconstructed phylogenetic trees from the following cancer patients: PRAD_0001204, PRAD_0001242, PRAD_0002273 and PRAD_0002898. Finally, Figure 4 consists of the reconstructed trees from PRAD_0005970, PRAD_0006108 and PRAD_0000377.

Part B

In Figure 5, we present the estimated posterior distribution of the number of subclones for IDC_0000525, IDC_0000690, LUAD_0000978, PRAD_0000655, PRAD_0003101 and PRAD_0003511. The estimated genotype and proportion matrices, conditioned on the estimated number of subclones, are in the main manuscript and S1 Tables. As a result of the resampling procedure employed at every step of the proposed algorithm, particles that best explain the input data are retained after resampling. In most cases, after a long iteration, the N particles for the genotype matrix are left with equal number of columns, which is returned as the number of subclones present in the tumor samples.

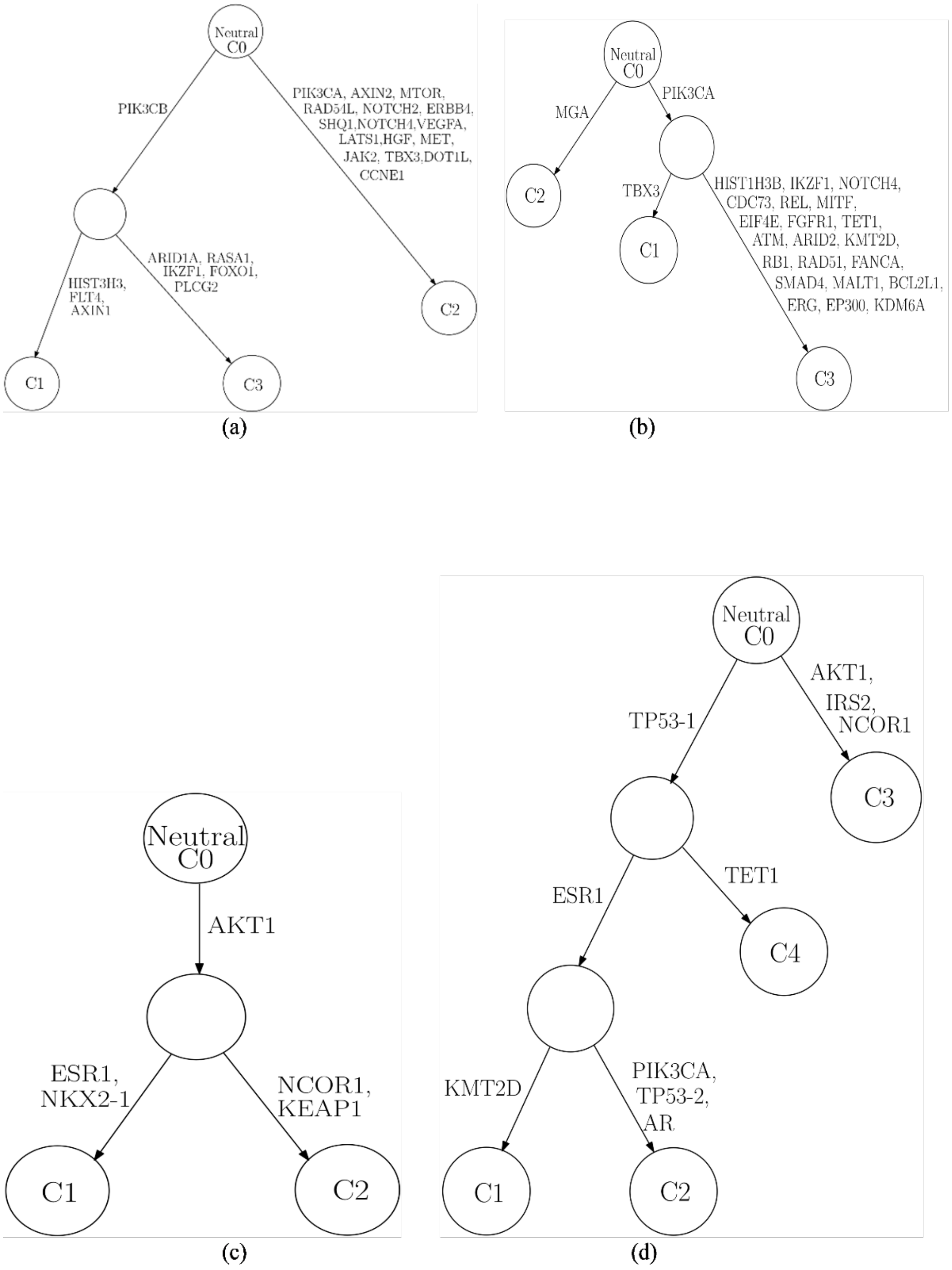

Figure 1. Constructed phylogenetic trees for other IDC patients. (a) IDC_0000247, (b) IDC_0002756 (c) IDC_0004183 and (d) IDC_0006161

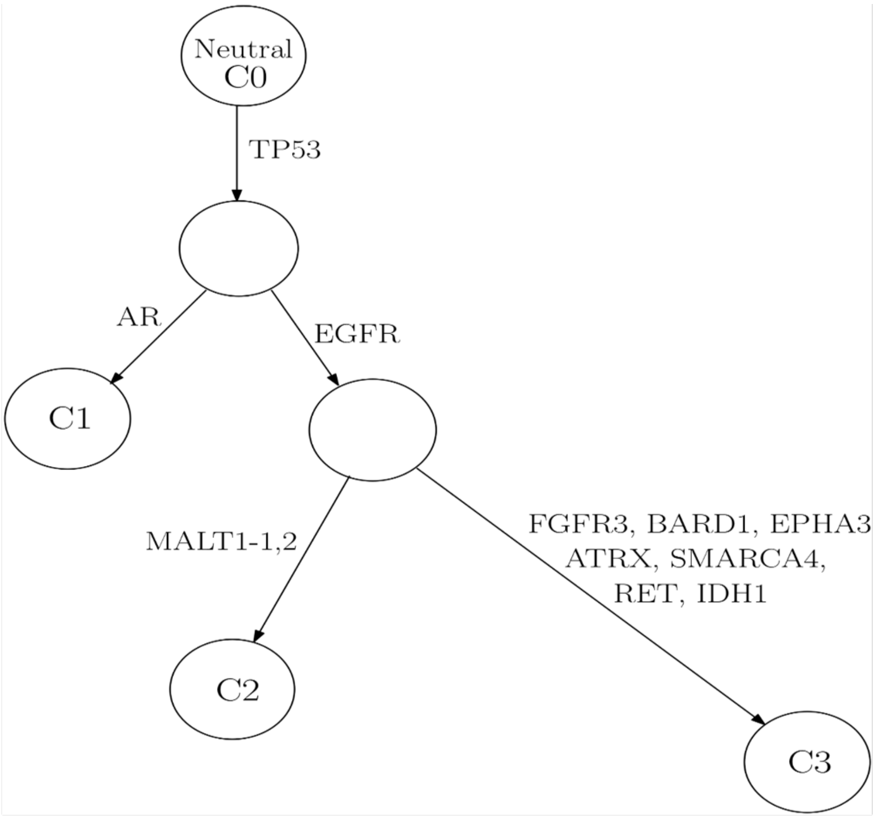

(a)

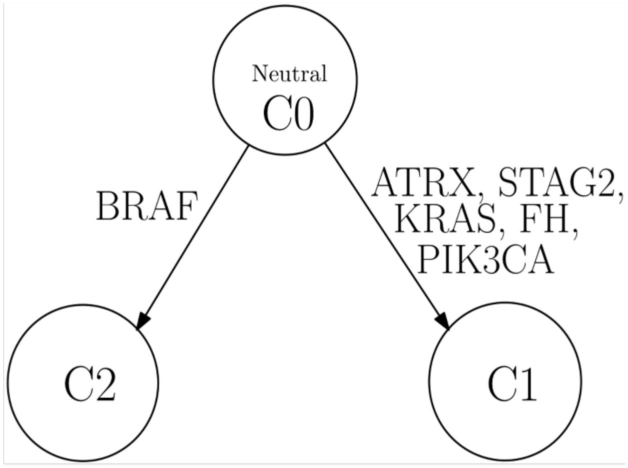

(b)

Figure 2. Constructed phylogenetic trees for other LUAD patients. (a) LUAD_0001303 and (b) LUAD_0012569

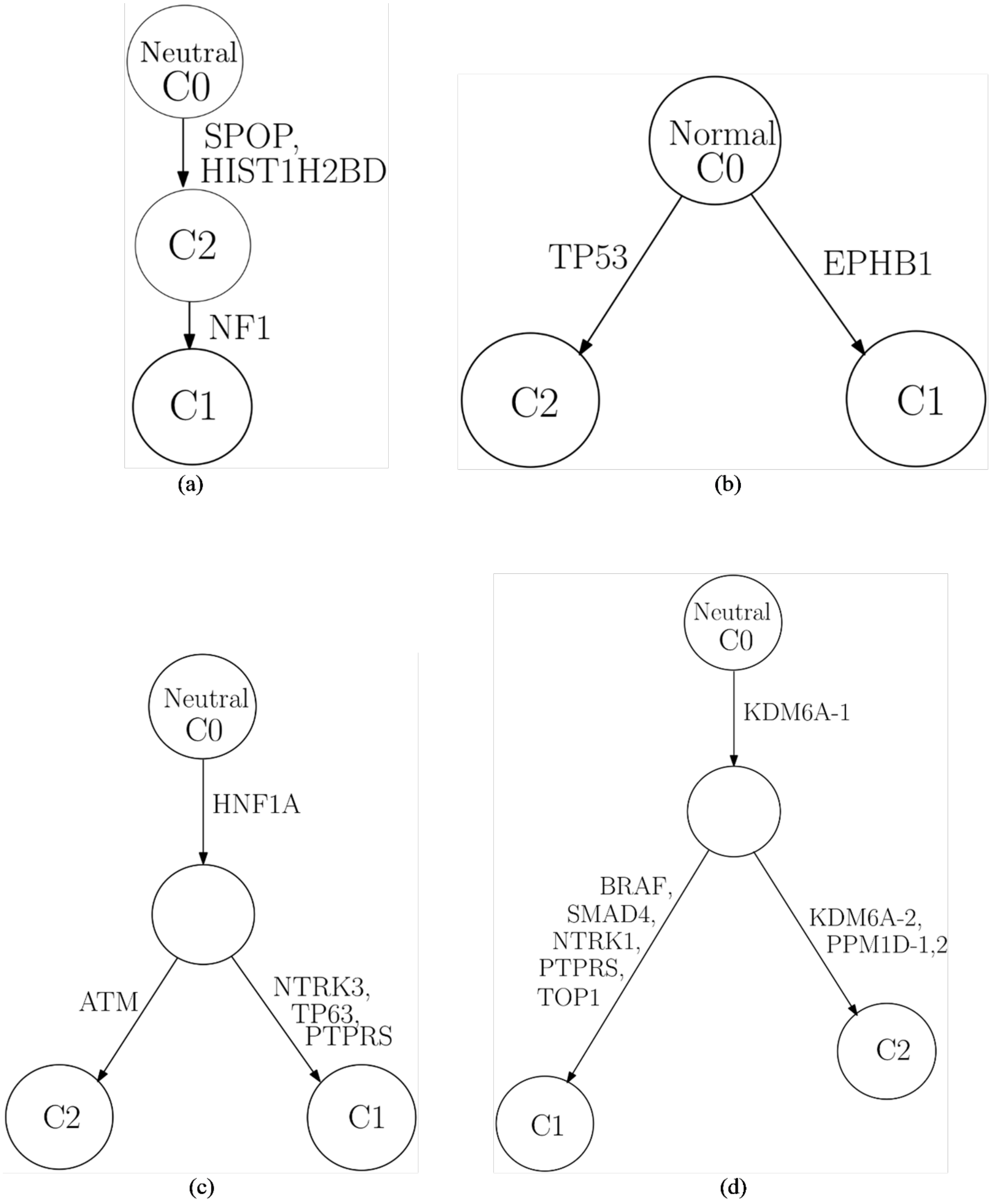

Figure 3. Constructed phylogenetic trees for other PRAD patients. (a) PRAD_0001204, (b) PRAD_0001242, (c) PRAD_0002273 and (d) PRAD_0002898

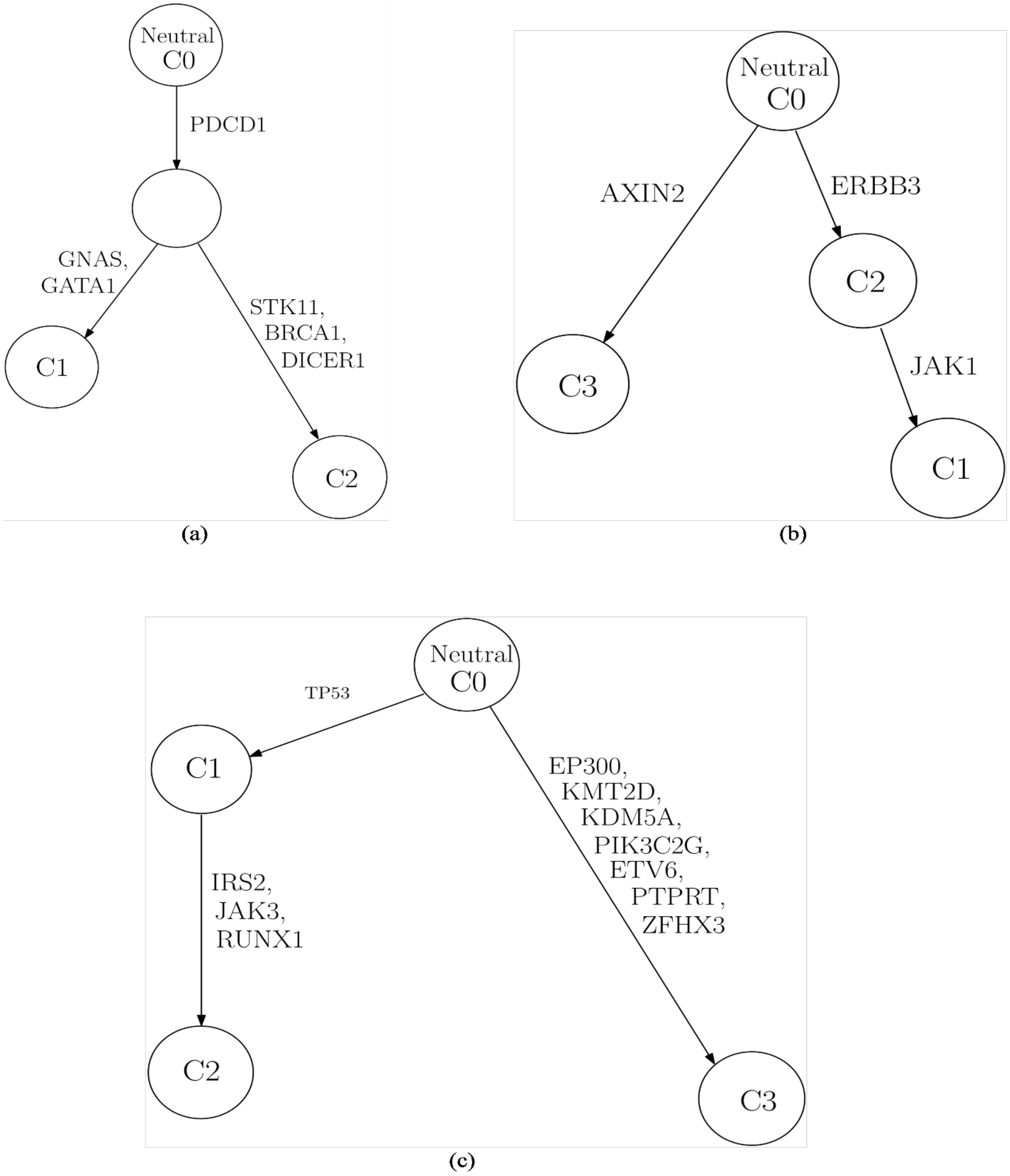

Figure 4. Constructed phylogenetic trees for other PRAD patients. (a) PRAD_0005970, (b) PRAD_0006108 and (c) PRAD_0000377.

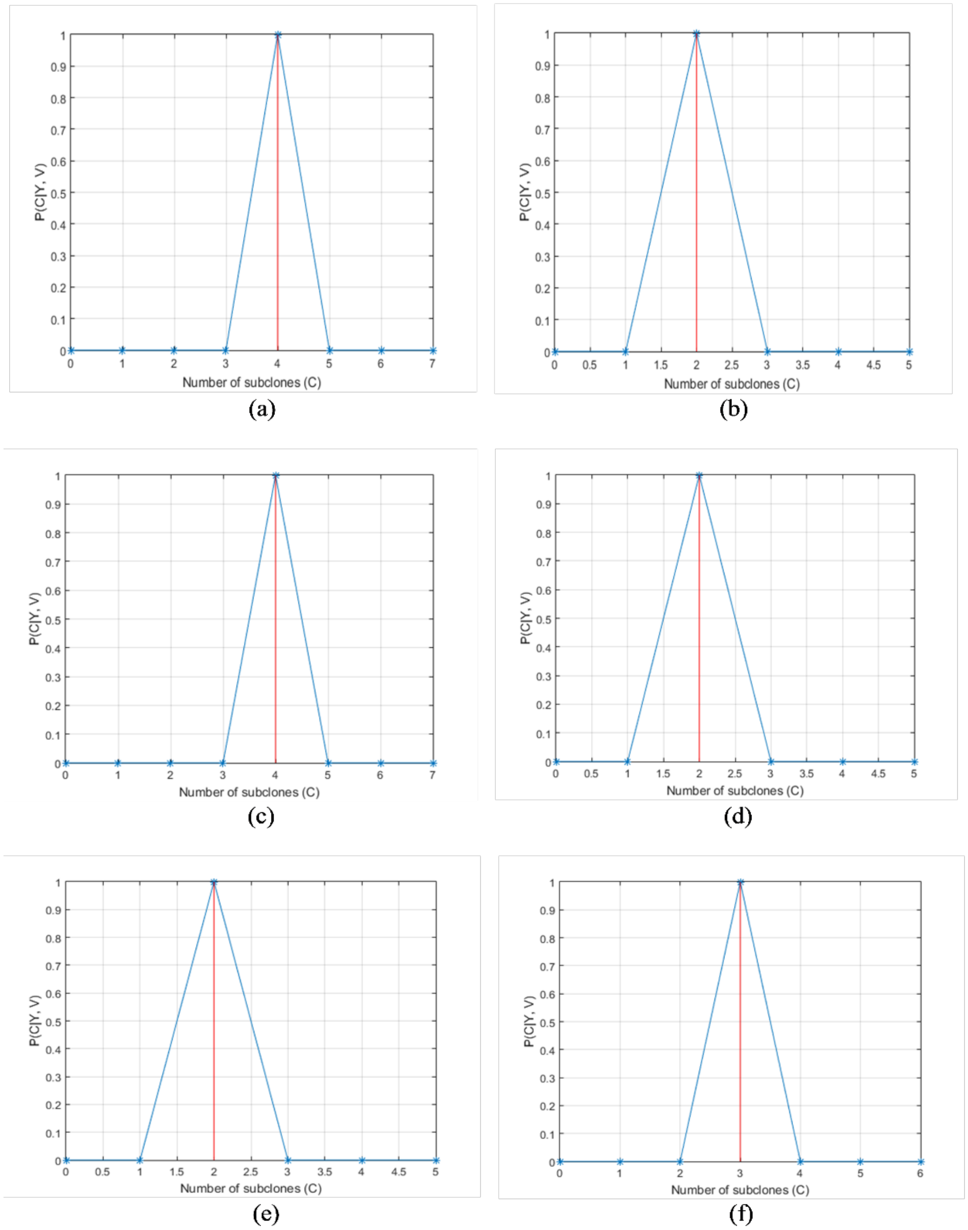

Figure 5. Posterior distributions for the number of subclones C in: (a) IDC_0000525, (b) IDC_0000690, (c) LUAD_0000978, (d) PRAD_0000655, (e) PRAD_0003101 and (f) PRAD_0003511.
