# Supplementary material for: A sequential Monte Carlo algorithm for inference of subclonal structure in cancer
Source: PLoS One. 2019 Jan 25;14(1):e0211213. doi: 10.1371/journal.pone.0211213 (PMC6347199; doi:10.1371/journal.pone.0211213)
Supplement: S1 Tables — Tables of the estimated genotypes of subclones. (PDF) [file pone.0211213.s002.pdf]

S1 Tables

Paper Title: A sequential Monte Carlo algorithm for inference of subclonal structure in cancer

Authors: Oyetunji Ogundijo , Kaiyi Zhu , Xiaodong Wang and Dimitris Anastassiou

Part A

In this section, we present the proportion matrices for the 3 cancer patients (IDC_0000525, IDC_0000690 and LUAD_0000978) presented in the main manuscript. Also, we present the genotype and proportion matrices for cancer patients: PRAD_0000655, PRAD_0003101 and PRAD_0003511 in Tables 2 - 4. In Table 1, the top block, the middle block and the bottom block contain the estimated proportion matrices from IDC_0000525, IDC_0000690 and LUAD_0000978, respectively. In Tables 2 - 4, the genotype matrices are in the top blocks and the proportion matrices are in the bottom blocks.

Part B

Here, we present the estimated genotype and proportion matrices for the remaining 13 cancer patients. Out of the 13 patients, there are 4 patients with IDC cancer type (IDC_0000247, IDC_0002756, IDC_0004183 and IDC_0006161), 2 patients with LUAD cancer type (LUAD_0001303 and LUAD_0012569) and 7 patients with PRAD cancer type (PRAD_0000377, PRAD_0001204, PRAD_0001242, PRAD_0002273, PRAD_0002898, PRAD_0005970 and PRAD_0006108). These results are presented in Figures 5 - 17. Each Figure consists of the genotype matrix (top block) and the proportion matrix (bottom block).

	Sample 1	Sample 2	Sample 3
C0	0.5758	0.7536	0.0043
C1	0.3158	0.0773	0.0316
C2	0.0000	0.0014	0.5567
C3	0.0000	0.0074	0.3564
C4	0.1084	0.1603	0.0510

	Sample 1	Sample 2	Sample 3
C0	0.0177	0.2079	0.0017
C1	0.5091	0.3623	0.3397
C2	0.4732	0.4298	0.6587

	Sample 1	Sample 2	Sample 3
C0	0.9490	0.0001	0.1637
C1	0.0041	0.3059	0.0006
C2	0.0068	0.0803	0.0001
C3	0.0402	0.6073	0.0000
C4	0.0000	0.0064	0.8356

Table 1: Estimated proportion matrices for IDC_0000525 (top), IDC_0000690 (middle) and LUAD_0000978 (bottom).

Gene name	C1	C2
CTNNB1	0	0 .5
KRAS	0.5	1
RYBP	0.5	1
SOX17	0	0 .5
FOXA1	0.5	1

	Sample 1	Sample 2	Sample 3
C0	0.9274	0.0005	0.9011
C1	0.0090	0.9913	0.0497
C2	0.0636	0.0082	0.0492

Table 2: Estimated genotypes (top) and estimated proportion (bottom) for PRAD_0000655

Gene name	C1	C2
PTEN	0.5	1
GRIN2A	0	0.5
PIK3CB	0	0.5
RB1	0	1
TP53	0	1

	Sample 1	Sample 2	Sample 3
C0	0.0048	0.0166	0.0000
C1	0.7071	0.0263	0.1407
C2	0.2880	0.9571	0.8593

Table 3: Estimated genotypes (top) and estimated proportion (bottom) for PRAD_0003101

Gene name	C1	C2	C3
TP53	0.5	0.5	1
TET2	0	0.5	0
CDK8	0.5	0.5	1
KDM6A	0	0	0.5
GATA3	0	0	0.5

	Sample 1	Sample 2	Sample 3
C0	0.4137	0.8605	0.1153
C1	0.2269	0.0882	0.8486
C2	0.3589	0.0011	0.0072
C3	0.0005	0.0502	0.0289

Table 4: Estimated genotypes (top) and estimated proportion (bottom) for PRAD_0003511

Gene name	C1	C2	C3
MSH6	0	0	0
SOX17	0	0	0
PIK3CA	0	0.5	0
PIK3CB	0.5	0	0.5
AXIN2	0	0.5	0
MTOR	0	0.5	0
RAD54L	0	0.5	0
NOTCH2	0	0.5	0
HIST3H3	0.5	0	0
ERBB4	0	0.5	0
SHQ1	0	0.5	0
FLT4	0.5	0	0
NOTCH4	0	0.5	0
VEGFA	0	0.5	0
LATS1	0	0.5	0
HGF	0	0.5	0
MET	0	0.5	0
JAK2	0	0.5	0
TBX3	0	0.5	0
AXIN1	0.5	0	0
DOT1L	0	0.5	0
CCNE1	0	0.5	0
ARID1A	0	0	0.5
RASA1	0	0	0.5
IKZF1	0	0	0.5
FOXO1	0	0	0.5
MGA	0	0	0
PLCG2	0	0	0.5

	Sample 1	Sample 2	Sample 3
C0	0.9601	0.0601	0.6625
C1	0.0000	0.3961	0.0789
C2	0.0000	0.5434	0.0002
C3	0.0399	0.0004	0.2584

Table 5: Estimated genotypes (top) and estimated proportion (bottom) for IDC_0000247

Gene name	C1	C2	C3
PIK3CA	0.5	0	0.5
TBX3	0.5	0	0
MGA	0	0.5	0
HIST1H3B	0	0	0.5
IKZF1	0	0	0.5
NOTCH4	0	0	0.5
CDC73	0	0	0.5
REL	0	0	0.5
MITF	0	0	0.5
EIF4E	0	0	0.5
FGFR1	0	0	0.5
TET1	0	0	0.5
ATM	0	0	0.5
ARID2	0	0	0.5
KMT2D	0	0	0.5
RB1	0	0	0.5
RAD51	0	0	0.5
FANCA	0	0	0.5
SMAD4	0	0	0.5
MALT1	0	0	0.5
BCL2L1	0	0	0.5
ERG	0	0	0.5
EP300	0	0	0.5
KDM6A	0	0	0.5

	Sample 1	Sample 2	Sample 3
C0	0.3916	0.1556	0.0000
C1	0.6029	0.2129	0.7408
C2	0.0006	0.5377	0.0001
C3	0.0049	0.0938	0.2591

Table 6: Estimated genotypes (top) and estimated proportion (bottom) for IDC_0002756

Gene name	C1	C2
AKT1	0.5	0.5
NCOR1	0	0.5
KEAP1	0	0.5
ESR1	0.5	0
NKX2-1	0.5	0

	Sample 1	Sample 2	Sample 3
C0	0.0003	0.0225	0.2386
C1	0.0004	0.9734	0.7612
C2	0.9992	0.0042	0.0002

Table 7: Estimated genotypes (top) and estimated proportion (bottom) for IDC_0004183

Gene name	C1	C2	C3	C4
PIK3CA	0	0.5	0	0
TP53	0.5	1	0	0.5
AKT1	0	0	0.5	0
PBRM1	0	0	0	0
TET1	0	0	0	0.5
IRS2	0	0	0.5	0
NCOR1	0	0	0.5	0
ESR1	0.5	0.5	0	0
KMT2D	0.5	0	0	0
AR	0	0.5	0	0

	Sample 1	Sample 2	Sample 3
C0	0.8112	0.4433	0.0721
C1	0.0010	0.0001	0.0962
C2	0.0000	0.0090	0.8291
C3	0.0021	0.4487	0.0026
C4	0.1856	0.0989	0.0000

Table 8: Estimated genotypes (top) and estimated proportion (bottom) for IDC_0006161

Gene name	C1	C2	C3
EGFR	0	0.5	0.5
FGFR3	0	0	0.5
AR	0.5	0	0
TP53	0.5	0	0.5
BARD1	0	0	0.5
EPHA3	0	0	0.5
ATRX	0	0	0.5
SMARCA4	0	0	0.5
RET	0	0	0.5
IDH1	0	0	0.5
MALT1	0	1	0

	Sample 1	Sample 2	Sample 3
C0	0.0001	0.1001	0.0000
C1	0.3919	0.3836	0.3686
C2	0.2399	0.2967	0.5754
C3	0.3682	0.2196	0.0560

Table 9: Estimated genotypes (top) and estimated proportion (bottom) for LUAD_0001303

Gene name	C1	C2
KRAS	0.5	0
PIK3CA	0.5	0
FH	0.5	0
ATRX	0.5	0
STAG2	0.5	0
BRAF	0	0.5

	Sample 1	Sample 2	Sample 3
C0	0.7282	0.9759	0.6399
C1	0.2346	0.0069	0.0021
C2	0.0372	0.0171	0.3580

Table 10: Estimated genotypes (top) and estimated proportion (bottom) for LUAD_0012569

Gene name	C1	C2	C3
TP53	0.5	0.5	0
EP300	0	0	0.5
KMT2D	0	0	0.5
KDM5A	0	0	0.5
PIK3C2G	0	0	0.5
ABL1	0	0	0
ETV6	0	0	0.5
IRS2	0	0.5	0
JAK3	0	0.5	0
RUNX1	0	0.5	0
APC	0	0	0
PTPRT	0	0	0.5
ZFHX3	0	0	0.5

	Sample 1	Sample 2	Sample 3	Sample 4	Sample 5
C0	0.0000	0.0004	0.0000	0.0171	0.8782
C1	0.6281	0.9976	0.5090	0.0250	0.0001
C2	0.0608	0.0001	0.4120	0.6107	0.0310
C3	0.3111	0.0020	0.0789	0.3473	0.0907

Table 11: Estimated genotypes (top) and estimated proportion (bottom) for PRAD_0000377

Gene name	C1	C2
SPOP	0.5	0 .5
HIST1H2BD	0.5	0 .5
NF1	0.5	0

	Sample 1	Sample 2	Sample 3
C0	0.8759	0.1548	0.0173
C1	0.0002	0.8452	0.9827
C2	0.1240	0.0000	0.0000

Table 12: Estimated genotypes (top) and estimated proportion (bottom) for PRAD_0001204

Gene name	C1	C2
TP53	0	0 .5
EPHB1	0.5	0

	Sample 1	Sample 2	Sample 3
C0	0.2077	0.0060	0.0389
C1	0.2480	0.2613	0.1241
C2	0.5443	0.7326	0.8370

Table 13: Estimated genotypes (top) and estimated proportion (bottom) for PRAD_0001242

Gene name	C1	C2
HNF1A	0.5	0 .5
ATM	0	0 .5
NTRK3	0.5	0
TP63	0.5	0
PTPRS	0.5	0

	Sample 1	Sample 2	Sample 3
C0	0.3619	0.0000	0.6860
C1	0.1229	0.1150	0.3116
C2	0.5152	0.8850	0.0024

Table 14: Estimated genotypes (top) and estimated proportion (bottom) for PRAD_0002273

Gene name	C1	C2
BRAF	0.5	0
SMAD4	0.5	0
KDM6A	0.5	1
NTRK1	0.5	0
PTPRS	0.5	0
TOP1	0.5	0
PPM1D	0	1

	Sample 1	Sample 2	Sample 3
C0	0.7524	0.0226	0.0000
C1	0.2338	0.9774	0.9871
C2	0.0139	0.0000	0.0129

Table 15: Estimated genotypes (top) and estimated proportion (bottom) for PRAD_0002898

Gene name	C1	C2
PDCD1	0.5	0.5
GNAS	0.5	0
GATA1	0.5	0
STK11	0	0.5
BRCA1	0	0.5
DICER1	0	0.5

	Sample 1	Sample 2	Sample 3
C0	0.0232	0.0059	0.0025
C1	0.5198	0.8784	0.3971
C2	0.4570	0.1157	0.6004

Table 16: Estimated genotypes (top) and estimated proportion (bottom) for PRAD_0005970

Gene name	C1	C2	C3
JAK1	0.5	0.5	1
ERBB3	0	0.5	0
AXIN2	0.5	0.5	1

	Sample 1	Sample 2	Sample 3
C0	0.4840	0.7121	0.8608
C1	0.4977	0.1783	0.0000
C2	0.0017	0.0080	0.1388
C3	0.0165	0.1015	0.0004

Table 17: Estimated genotypes (top) and estimated proportion (bottom) for PRAD_0006108
