# Supplementary material for: A sequential Monte Carlo algorithm for inference of subclonal structure in cancer
Source: PLoS One. 2019 Jan 25;14(1):e0211213. doi: 10.1371/journal.pone.0211213 (PMC6347199; doi:10.1371/journal.pone.0211213)
Supplement: S1 File — Detailed description of the sampling procedure from the prior distribution of a ternary matrix using the cIBP, sequential construction of a ternary matrix, and the detailed description of our proposed SMC algorithm. (PDF) [file pone.0211213.s003.pdf]

S1 File

Oyetunji Ogundijo , Kaiyi Zhu , Xiaodong Wang and Dimitris Anastassiou

S1

State-Space Formulation

Our state-space formulation of the model presented in the main manuscript exploits the sequential construction of the ternary matrix $\mathbf{Z}$ (discussed below). Specifically, we consider the tth row of the data matrix $\mathbf{Y}$ and $\mathbf{V}$ as the new observation at *time* t of our state-space model, treat the tth row of the ternary matrix $\mathbf{Z}$ as the hidden state at *time* t, and $\mathbf{W}$ and p as the parameters of the model. Before explicitly stating the state transition and the observation models, we describe the prior distribution of the general $(Q+1)$-nary matrix $\mathbf{Z}$, with dimension $T \times C$ [6, 5], given by:

$$p(\mathbf{Z}) = \left(\frac{1}{\prod_t^T (t + \alpha/C)}\right)^C \prod_{c=1}^{C_+} \left(\frac{\alpha}{C} \cdot \frac{1}{Q}\right) \frac{(T - m_c)!}{T!} \times \prod_{j=1}^{m_c-1} \left[\frac{(j + \alpha/C)}{(j + Q\beta)}\right] \frac{1}{\beta} \prod_{q=1}^{Q} \frac{\Gamma(\beta + m_{cq})}{\Gamma(\beta)}, \quad (1)$$

where C_+ denotes the number of columns of $\mathbf{Z}$ with non-zero entries, $m_{cq} = \sum_{t=1}^{T} \mathbb{I}(z_{tc} = q)$ denotes the number of rows possessing value $q \in \{1, 2, ..., Q\}$ in column c, and $m_c = \sum_{q=1}^{Q} m_{cq}$. However, as $C \to \infty$, [6] showed that the resulting distribution of $\mathbf{Z}$ is the categorical Indian buffet process (cIBP), i.e., $\mathbf{Z} \sim \text{cIBP}_C(Q, \alpha, \beta)$, a sequential process for constructing a $(Q+1)$-nary matrix with a known number of rows and an unknown number of columns.

In our case, we consider a special case of cIBP, i.e., $\mathbf{Z} \sim \text{cIBP}_C(Q = 2, \alpha, \beta)$ and next discuss its *sequential generative process* [6, 5], a direct generalization of the Indian Buffet Process (IBP) [7]. Imagine that in an Indian buffet restaurant, there is an infinite number of dishes and assume that each dish comes with 2 choices, i.e.,. $Q = 2$. For instance, assuming that spice level of any particular choice of dish can be normal or hot. Now, we have T customers who arrive at the restaurant sequentially, one after the other. The first customer walks into the restaurant and loads her plate from the first c_1 dishes, where $c_1 = \text{Pois}(\alpha)$, and the spice level of the food in each of the dishes in the c_1 plates is chosen with probability $\beta/\beta^\star$, where $\beta^\star = 2\beta$. The tth customers will choose a particular dish with a specific choice according to the popularity of the dish, i.e., choosing

a dish c with a particular spice level with probability $\left(\frac{m_c^t}{t}\right) \times \left(\frac{\beta + m_{cq}^t}{\beta^* + m_c^t}\right)$, where $m_{cq}^t = \sum_{j=1}^{t-1} \mathbb{I}(z_{jc} = q)$, $q \in \{1, 2\}$ denotes the number of customers who have tasted dish c before customer t with spice level q and $m_c^t = \sum_{q=1}^{2} m_{cq}^t$ denotes the total number of customers before customer t who have tasted dish c. In addition, customer t chooses $\text{Pois}\left(\frac{\alpha}{t}\right)$ new dishes, with the spice level of each of the chosen new dishes determined with probability β/β^*.

Now, if we record the choices of each customer on each row of a matrix, where each column corresponds to a dish on the buffet (0.5 or 1, depending on the spice level if the dish is chosen, and 0 if not), then such a ternary matrix is a draw from $\text{cIBP}_C(Q, \alpha, \beta)$ [6, 5], with $Q = 2$. The entire process is sequential because the choices made by the tth customer are dependent only on the choices made by the $t-1$ preceding customers and not on the remaining $T-t$ customers.

In our case, the dishes in the cIBP are the distinct subclones in the tumor samples, the SNVs are the customers and more importantly, the tth customer is the observation at *time* t in our state-space model. Moreover, if we consider $\mathbf{z}_t = [z_{t1}, z_{t2}, ..., z_{tC}]$ the tth row of $\mathbf{Z}$ as the state at time t, then we can write our state transition model, following the sequential process described by the cIBP as follows:

$$P(\mathbf{z}_t | \mathbf{Z}_{t-1}, \alpha, \beta), \tag{2}$$

where $\mathbf{Z}_{t-1}$ denotes the previous $t-1$ rows in $\mathbf{Z}$, α and β are positive real numbers. The algorithm to sample from (2) is presented in **Algorithm 1** in the main manuscript. Note that in **Algorithm 1**, $\mathbf{Z}_t$ is implicitly constructed from $\mathbf{Z}_{t-1}$ and if in the process, new non-zero column(s) is/are introduced in $\mathbf{Z}_t$ ($\text{Pois}(\alpha/t) > 0$), then new row(s) will be added to $\mathbf{W}$ as well. On the other hand, if the numbers of non-zero columns in $\mathbf{Z}_{t-1}$ and $\mathbf{Z}_t$ are the same, then the number of rows in $\mathbf{W}$ does not change between $t-1$ and t. To account for any possible change of dimension in $\mathbf{W}$, we re-parameterize matrix $\mathbf{W}$. Specifically, we rewrite $w_{cs} = \theta_{cs} / \sum_{c'=0}^{C} \theta_{c's}$, which implies that we estimate θ_{cs} and compute w_{cs} from the estimates of θ_{cs}. This procedure ensures that each column of $\mathbf{W}$ sums to unity at any point in time during the process.

Moreover, since we are interested in the final estimates of the model parameters $\mathbf{W}$ and p, we create artificial dynamics for these parameters using the random walk model, i.e.,

$$\begin{aligned} \phi_t &\sim p(\phi_t | \phi_{t-1}) = \mathcal{N}(\phi_{t-1}, \sigma^2), \\ \phi_t &\in \{p, \theta_{cs}, c = 0, 1, ..., C, s = 1, ..., S\}, \end{aligned} \tag{3}$$

where σ denotes the standard deviation. Hence, (2)-(3) fully describe the system state transition.

Similarly, the observation at time t is given by:

$$\begin{aligned} \mathbf{y}_t \sim P(\mathbf{y}_t | \mathbf{Z}_{1:t}, \mathbf{W}, p) &= P(\mathbf{y}_t | \mathbf{z}_t, \mathbf{W}, p) \\ &= \prod_{s=1}^{S} \text{Binomial}(y_{ts} | v_{ts}, p_{ts}), \end{aligned} \tag{4}$$

where $\mathbf{y}_t$ denotes the observation at time t (which is conditionally independent of the previous observations $\mathbf{Y}_{t-1}$ given the state $\mathbf{z}_t$), i.e., the tth row of $\mathbf{Y}$. (4) fully describes the measurement model for the system. Finally, (2) - (4) completely describe our proposed state-space model for estimating C, $\mathbf{Z}$, $\mathbf{W}$ and p from the observed VAFs of SNVs obtained from NGS.

The SMC Algorithm

We describe in greater details the SMC filtering framework that we employed to estimate the states and the parameters of our proposed state-space model [9]. Consider the general dynamic system with hidden state variable $\mathbf{x}_t$, in our case, consisting of categorical variables $\mathbf{z}_t$ and continuous variables ϕ_t, $\phi_t \in \{p_0^t, \theta_{cs}^t, c = 0, 1, ..., C, s = 1, ..., S\}$, and measurement variable $\mathbf{y}_t$, where there is an initial state model $p(\mathbf{x}_0)$, and $\forall t \geq 1$, a state transition model given in (2) - (3) and an observation model given in (4). The sequence $\mathbf{X}_t = \{\mathbf{x}_1, \mathbf{x}_2, ..., \mathbf{x}_t\}$ is not observed and we want to estimate it for each time t, given that the we have the observations $\mathbf{Y}_t = \{\mathbf{y}_1, \mathbf{y}_2, ..., \mathbf{y}_t\}$.

Our goal is to approximate the posterior distribution of states $p(\mathbf{X}_t|\mathbf{Y}_t)$ using samples drawn from it. However, getting such samples from $p(\mathbf{X}_t|\mathbf{Y}_t)$ is usually not feasible. We can still implement an estimate using N samples, $\{\mathbf{X}_t^i\}_{i=1}^N$, taken from another distribution, $q(\mathbf{X}_t|\mathbf{Y}_t)$, whose support includes the support of $p(\mathbf{X}_t|\mathbf{Y}_t)$ (importance sampling theorem). For the approximation, the weights associated with the samples are calculated as follows:

$$\tilde{w}_t^i = \frac{p(\mathbf{X}_t|\mathbf{Y}_t)}{q(\mathbf{X}_t|\mathbf{Y}_t)} \text{ and } w_t^i = \frac{\tilde{w}_t^i}{\sum_{m=1}^N \tilde{w}_t^m},\ i = 1, ..., N. \tag{5}$$

Thus, the pair $\{\mathbf{X}_t^i, w_{1:t}^i\}_{i=1}^N$ is said to be properly weighted with respect to the distribution $p(\mathbf{X}_t|\mathbf{Y}_t)$, and the approximation $\hat{p}(\mathbf{X}_t|\mathbf{Y}_t)$ is then given by:

$$\hat{p}(\mathbf{X}_t|\mathbf{Y}_t) = \sum_{i=1}^N w_t^i \delta(\mathbf{X}_t - \mathbf{X}_t^i), \text{ where } \delta(\mathbf{u}) = \begin{cases} 1, & \text{if } \mathbf{u} = \underline{\mathbf{0}} \\ 0, & \text{otherwise.} \end{cases} \tag{6}$$

Similar to the above importance sampling theory, a sequential algorithm can be obtained as follows. First, we express the full posterior distribution of states $\mathbf{X}_t$ given the observations $\mathbf{Y}_t$ as follows:

$$\begin{aligned} p(\mathbf{X}_t|\mathbf{Y}_t) &\propto p(\mathbf{y}_t|\mathbf{X}_t, \mathbf{Y}_{t-1})p(\mathbf{X}_t|\mathbf{Y}_{t-1}) \\ &= p(\mathbf{y}_t|\mathbf{X}_t, \mathbf{Y}_{t-1})p(\mathbf{x}_t|\mathbf{X}_{t-1}, \mathbf{Y}_{t-1})p(\mathbf{X}_{t-1}|\mathbf{Y}_{t-1}). \end{aligned} \tag{7}$$

At time t, we desire to obtain N weighted samples from $p(\mathbf{X}_t|\mathbf{Y}_t)$, which is not feasible. Instead, we define an importance distribution $q(\mathbf{X}_t|\mathbf{Y}_t) = q(\mathbf{x}_t|\mathbf{X}_{t-1}, \mathbf{Y}_t)q(\mathbf{X}_{t-1}|\mathbf{Y}_{t-1})$, from which the samples can be obtained from, and then calculate the associated unnormalized importance weights as follows:

$$\tilde{w}_t^i = \frac{p(\mathbf{y}_t|\mathbf{X}_t^i, \mathbf{Y}_{t-1})p(\mathbf{x}_t^i|\mathbf{X}_{t-1}^i, \mathbf{Y}_{t-1})}{q(\mathbf{x}_t^i|\mathbf{X}_t^i, \mathbf{Y}_t)} \frac{p(\mathbf{X}_{t-1}^i|\mathbf{Y}_{t-1})}{q(\mathbf{X}_{t-1}^i|\mathbf{Y}_{t-1})}. \tag{8}$$

Assuming that at time $t-1$, we have already drawn the samples $\{\mathbf{X}_{t-1}^i\}_{i=1}^N$ from the importance distribution $q(\mathbf{X}_{t-1}|\mathbf{Y}_{t-1})$ and the corresponding normalized weights written as follows:

$$w_{t-1}^i \propto \frac{p(\mathbf{X}_{t-1}^i|\mathbf{Y}_{t-1})}{q(\mathbf{X}_{t-1}^i|\mathbf{Y}_{t-1})}, \quad i = 1, ..., N, \tag{9}$$

we can now draw samples $\{\mathbf{X}_t^i\}_{i=1}^N$ from the importance distribution $q(\mathbf{X}_t|\mathbf{Y}_t)$ by drawing the new state samples for the time step t as $\mathbf{x}_t^i \sim q(\mathbf{x}_t|\mathbf{X}_{t-1}^i, \mathbf{Y}_t)$, and write $\{\mathbf{X}_t^i\}_{i=1}^N = \{\mathbf{x}_t^i, \mathbf{X}_{t-1}^i\}_{i=1}^N$. If we substitute (9) into (8), the weights at time t satisfy the recursion:

$$\tilde{w}_t^i \propto w_{t-1}^i \frac{p(\mathbf{y}_t|\mathbf{X}_t^i, \mathbf{Y}_{t-1})p(\mathbf{x}_t^i|\mathbf{X}_{t-1}^i, \mathbf{Y}_{t-1})}{q(\mathbf{x}_t^i|\mathbf{X}_t^i, \mathbf{Y}_t)}, \quad i = 1, ..., N, \tag{10}$$

and then the weights are normalized to sum to unity.

So far, we have presented a generic sequential sampling algorithm. We obtain the optimal importance distribution by setting $q(\mathbf{x}_t^i|\mathbf{X}_{t-1}^i, \mathbf{Y}_t) = p(\mathbf{x}_t^i|\mathbf{X}_{t-1}^i, \mathbf{Y}_t)$, and the weights in (10) becomes $\tilde{w}_t^i \propto w_{t-1}^i p(\mathbf{y}_t|\mathbf{X}_{t-1}^i, \mathbf{Y}_{t-1})$ [8] i.e., if the distributions $p(\mathbf{y}_t|\mathbf{X}_t^i, \mathbf{Y}_{t-1})$ and $p(\mathbf{x}_t^i|\mathbf{X}_{t-1}^i, \mathbf{Y}_{t-1})$ are conjugates, then closed form solutions can be obtained for $p(\mathbf{x}_t^i|\mathbf{X}_{t-1}^i, \mathbf{Y}_t)$, and hence, $p(\mathbf{y}_t|\mathbf{X}_{t-1}^i, \mathbf{Y}_{t-1})$. However, if no such conjugacy exists, which is the case for our state-space model, the most popular choice and equally efficient solution is to set $q(\mathbf{x}_t^i|\mathbf{X}_{t-1}^i, \mathbf{Y}_t) = p(\mathbf{x}_t^i|\mathbf{X}_{t-1}^i)$ (in (2)-(3)) [10]. Considering the assumed independence in our model, i.e., $p(\mathbf{x}_t^i|\mathbf{X}_{t-1}^i, \mathbf{Y}_{t-1}) = p(\mathbf{x}_t^i|\mathbf{X}_{t-1}^i)$ and $p(\mathbf{y}_t|\mathbf{X}_t^i, \mathbf{Y}_{t-1}) = p(\mathbf{y}_t|\mathbf{x}_t^i)$, then (10) becomes:

$$\begin{aligned} \tilde{w}_t^i &\propto w_{t-1}^i p(\mathbf{y}_t|\mathbf{x}_t^i) \\ &= w_{t-1}^i p(\mathbf{y}_t|\mathbf{z}_t^i, \mathbf{W}_t^i), \end{aligned} \tag{11}$$

and the weights are normalized. Such implementation is commonly referred to as a bootstrap filter in the literature [10].

However, the variance of the weights increases over time, a condition referred to as degeneracy in the literature [9]. To avoid this, we perform resampling, at every time step, owing to the choice of the importance distribution [10], discarding the ineffective samples and multiplying the effective ones. The resampling procedure [11] is briefly summarized as follows:

- Interpret each weight w_t^i as the probability of obtaining the sample index i.
- Draw N samples from the discrete probability distribution $\{w_t^i\}$ and replace the old sample set with this new one.
- Set all weights to the constant value $w_t^i = 1/N$.

Finally, our proposed SMC algorithm for estimating the states and the parameters of our state-space model is presented in **Algorithm 2** in the main

manuscript. The algorithm is initialized by taking samples from the prior distributions of the parameters. We assume the following:

$$\begin{aligned}
\theta_{0s} &\overset{i.i.d}{\sim} \text{Gamma}(a_0, 1),\ s = 1, ..., S, \\
\theta_{cs} &\overset{i.i.d}{\sim} \text{Gamma}(a_1, 1),\ s = 1, ..., S, c = 1, ..., C,\ \text{and} \\
p &\sim \text{Beta}(a_{00}, b_{00}),
\end{aligned} \tag{12}$$

such that $w_{cs} = \theta_{cs} / \sum_{c'=0}^{C} \theta_{c's}$ and consequently, $\sum_{c'=0}^{C} w_{c's} = 1$. We report the posterior estimates of all the unknown variables using the procedure described below.

S2

Here, we present how the point estimates of the unknown variable are obtained from the posterior weighted Monte Carlo samples following the procedures highlighted in [2].

First, we factorize the joint posterior distribution of all the unknown variables given the input dataset (matrices $\mathbf{Y}$ and $\mathbf{V}$) as follows:

$$p(C, \mathbf{Z}, \mathbf{W}, p|\mathbf{Y}, \mathbf{V}) = p(C|\mathbf{Y}, \mathbf{V})p(\mathbf{Z}|\mathbf{Y}, \mathbf{V}, C)p(\mathbf{W}, p|\mathbf{Y}, \mathbf{V}, C, \mathbf{Z}).$$

Then, based on the available posterior Monte Carlo samples for $\mathbf{Z}$, we approximately evaluate the marginal posterior distribution for C (the number of columns of N particles of the genotype matrices) and then determine the maximum a posteriori (MAP) estimate $\hat{C}$.

Next, conditional on $\hat{C}$, we then estimate $\mathbf{Z}$ as follows. For any two matrices $\mathbf{Z}$ and $\mathbf{Z}'$, $1 \le c, c' \le \hat{C}$, define

$$\mathcal{D}_{cc'}(\mathbf{Z}, \mathbf{Z}') = \sum_{t=1}^{T} |z_{tc} - z'_{tc'}|,$$

and define a distance

$$d(\mathbf{Z}, \mathbf{Z}') = \min \sum_{c=1}^{\hat{C}} \mathcal{D}_{c,\pi_c}(\mathbf{Z}, \mathbf{Z}'),$$

where π_c is a permutation of $\{1, ..., \hat{C}\}$ and the minimum is over all possible permutations. Thus, an estimate for $\mathbf{Z}$ is defined as:

$$\begin{aligned}
\hat{\mathbf{Z}} &= \operatorname*{argmin}_{\mathbf{Z}'} \int d(\mathbf{Z}, \mathbf{Z}') dp(\mathbf{Z}|\mathbf{Y}, \hat{C}) \\
&\approx \operatorname*{argmin}_{\mathbf{Z}'} \sum_{i=1}^{N} w_T^i d(\mathbf{Z}_T^i, \mathbf{Z}')
\end{aligned}$$

for posterior Monte Carlo samples, $\{\mathbf{Z}_T^i\}_{i=1}^N$ and the normalized weights $\{w_i\}_{i=1}^N$.

Finally, we report posterior estimates $\hat{\mathbf{W}}$ and $\hat{p}$ for $\mathbf{W}$ and p, respectively conditional on $\hat{C}$ and $\hat{\mathbf{Z}}$.

S3

In our experiments, we set $N \leq 1000$, $a_0 = 0.1$, $a = 8$, $a_{00} = 1$, and $b_{00} = 20$. $\beta = 2$ can be any integer below 50 and we set $\beta = 2$. Similarly, we set α between 0.01 and 1 for the simulated and the real tumor datasets, respectively. For the experiments involving Bayclone, we ran $30,000$ iterations, discarding the first $15,000$ as burn-in, and thinning the chain by taking every 30th sample.

References

[1] Jiao,W., et al. (2014) Inferring clonal evolution of tumors from single nucleotide somatic mutations. *BMC bioinformatics*, **15**, 35.

[2] Lee,J., et al. (2015) A Bayesian feature allocation model for tumor heterogeneity. *The Annals of Applied Statistics*, **9**, 621–639.

[3] Schuh,A., et al. (2012) Monitoring chronic lymphocytic leukemia progression by whole genome sequencing reveals heterogeneous clonal evolution patterns. *Blood*, **120**, 4191–4196.

[4] Zare,H., et al. (2014) Inferring clonal composition from multiple sections of a breast cancer. *PLoS computational biology*, **10**, e1003703.

[5] Sengupta,S., et al. (2015) Bayclone: Bayesian nonparametric inference of tumor subclones using NGS data. *In: Pacific Symposium on Biocomputing*, **20**, 2015. p. 467.

[6] Sengupta,S. (2013) Two models involving Bayesian nonparametric techniques. *University of Florida.*

[7] Griffiths,T.L. and Ghahramani, Z. (2011) The indian buffet process: An introduction and review. *Journal of Machine Learning Research*, **12**, 1185–1224.

[8] Jajamovich,G., et al. (2011) Bayesian multiple-instance motif discovery with BAMBI: inference of recombinase and transcription factor binding sites. *Nucleic acids research*, **39**, e146–e146.

[9] Doucet,A., et al. (2001) Sequential Monte Carlo methods in practice Springer. *New York.*

[10] Särkkä,S. *et al.* (2013) Particle filtering. *Bayesian filtering and smoothing*, Cambridge University Press, Vol. 3, pp. 129–132.

[11] Arulampalam,M. et al. (2002) A tutorial on particle filters for online nonlinear/non-Gaussian Bayesian tracking. *IEEE Transactions on signal processing*, **50**, 174–188.
